# Recent Advances in Chinese Herbal Medicine for Cerebral Ischemic Reperfusion Injury

**DOI:** 10.3389/fphar.2021.688596

**Published:** 2022-01-17

**Authors:** Ping Huang, Haitong Wan, Chongyu Shao, Chang Li, Ling Zhang, Yu He

**Affiliations:** ^1^ School of Pharmaceutical Sciences, Zhejiang Chinese Medical University, Hangzhou, China; ^2^ School of Life Sciences, Zhejiang Chinese Medical University, Hangzhou, China

**Keywords:** Chinese herbal medicine, cerebral ischemia, reperfusion injury, pathophysiology, pharmacological effects, mechanisms

## Abstract

Cerebral ischemic reperfusion injury (CI/RI) is a critical factor that leads to a poor prognosis in patients with ischemic stroke. It is an extremely complicated pathological process that is clinically characterized by high rates of disability and mortality. Current available treatments for CI/RI, including mechanical and drug therapies, are often accompanied by significant side effects. Therefore, it is necessary to discovery new strategies for treating CI/RI. Many studies confirm that Chinese herbal medicine (CHM) was used as a potential drug for treatment of CI/RI with the advantages of abundant resources, good efficacy, and few side effects. In this paper, we investigate the latest drug discoveries and advancements on CI/RI, make an overview of relevant CHM, and systematically summarize the pathophysiology of CI/RI. In addition, the protective effect and mechanism of related CHM, which includes extraction of single CHM and CHM formulation and preparation, are discussed. Moreover, an outline of the limitations of CHM and the challenges we faced are also presented. This review will be helpful for researchers further propelling the advancement of drugs and supplying more knowledge to support the application of previous discoveries in clinical drug applications against CI/RI.

## 1 Introduction

Cerebral ischemia (CI), a universal cerebrovascular disease with intracranial ischemia and hypoxia, often occurs in stroke or “brain attack” and results in local tissue necrosis and secondary apoptosis. Ischemic stroke accounts for 85% of all strokes, and hemorrhagic stroke accounts for 15% (Rai et al., 2016). It is characterized by sudden onset, high risk of disease, high mortality rate, and long-term disability, which brings a severe burden to the normal life of individuals and the cost of the medical care system (Liu R. et al., 2020). With about 15 million cases occurring every year (Manwani and McCullough, 2013) and a 30% mortality rate, CI is regarded as the third leading cause of death and a major cause of disability worldwide (Wang R. et al., 2019). The patients of CI should be diagnosed rapidly and treated promptly and appropriately; otherwise, it is very difficult to cure.

Saving the damaged neurons and recovering the impaired neuronal function are two main measures for treating ischemic stroke clinically. Most ischemic stroke survivors suffer irreversible neurological damage that worsens their quality of life. Currently, magnetic resonance imaging and cranial computed tomography (CT) are used only for diagnosis, whereas patients need effective drug intervention at once to achieve a good prognosis. Up to now, intravenous thrombolysis with Alteplase (rt-PA) is the only FDA-approved treatment for ischemic stroke, which carries a restrictive therapeutic window within 4.5 h to achieve the treatment of ischemic stroke (Catanese et al., 2017). There is no virtual progress in the treatment of CI from only a limited number of available drugs in recent years. Once CI occurs, restoration of blood oxygen supply in time is the first principle of current clinical treatment, and the patients suffer the injury and cascade reactions caused by CI and reperfusion. CI reperfusion injury (CI/RI), a complicated pathological process, usually happens in succession with CI. Therefore, it is necessary to develop new drugs or therapies to lengthen the therapeutic window and ameliorate the consequences of CI/RI.

The pathological mechanism of CI/RI is extremely complex and involves many factors, such as energy metabolism disorders, Ca^2+^ overload, inflammatory and oxidative stress, excitotoxicity, ferroptosis, apoptosis (Auriel and Bornstein, 2010), and some novel forms of programmed cell death. Autophagy, ferroptosis, parthanatos, and pyroptosis, as the novel forms of programmed cell death, have attracted much attention in recent years. During reperfusion, CI is only partially salvaged, but further damages flood in. The procedure of injury and remedy in the brain may interlace together, which makes it extremely difficult to develop effective treatments for CI/RI with such complex factors and their interactions. In conclusion, the prevention of CI/RI can decrease its incidence and mortality.

**FIGURE 1:**
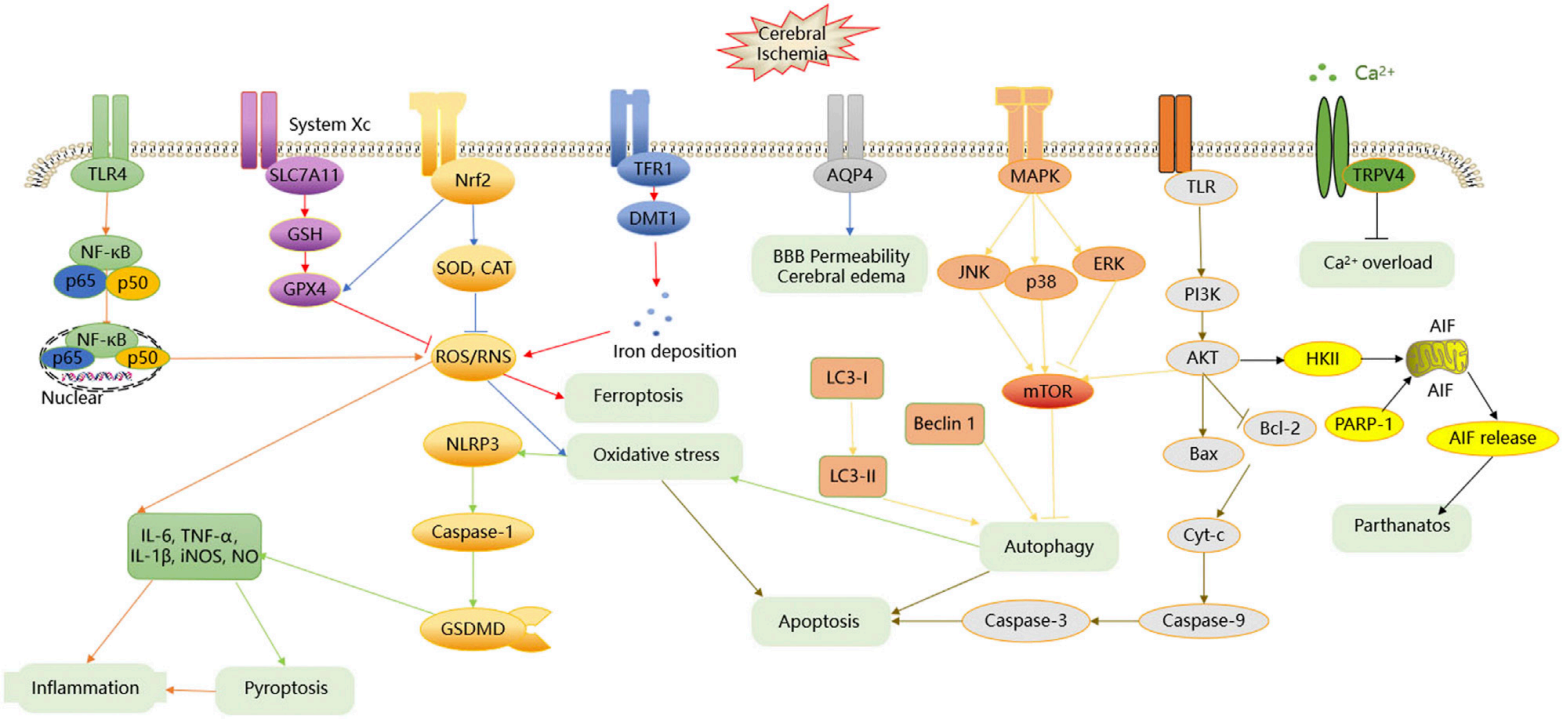
The effects and mechanisms of CI/RI.

Chinese herbal medicine (CHM) maintains an irreplaceable position in the health system all the time in China. Recently, it has also been recognized as a fertile source of novel lead molecules for modern drug discovery in other countries (Long et al., 2012). With the increasing trend of CI/RI, more and more researchers have fixed their eyes on exploring CHM, which has the characteristics of multi-ingredient, multitarget, and multipathway to work together. CHM functions holistically and shows an obvious superiority over “one drug, one target” treatment, especially in treating multifactorial diseases, such as CI/RI. Routinely, CHM has many therapeutic effects for CI/RI, such as antioxidation; anti-inflammation; antiapoptosis; promotion of angiogenesis; enhancement of autophagy; adjustment of energy metabolism; protection of blood–brain barrier (BBB) integrity; prevention of Ca^2+^ overload; and regulation of the neurological cell proliferation, differentiation, and regeneration. The latest studies are making progress in borneol intervention (Li et al., 2021), polysaccharide intervention (Meng et al., 2021) for CI/RI, and reviewing phytochemicals as regulators of microglia/macrophage activation in CI/RI (Subedi and Gaire, 2021). Li *et al.* and Meng *et al.* comprehensively highlight the potential application of borneol and polysaccharides as a neuroprotective agent against CI/RI. Subedi *et al.* makes significant progress in the study of phytochemicals and their derivatives as promising drug candidates for the treatment of tissue inflammatory injury. This review article focuses on the role of extraction components of single CHM, CHM formulas, and CHM preparations that have protective effects in animal and cell CI/RI models by multiple mechanisms. To explore the complex mechanism of CHM against CI/RI, we summarize the effects of the CHM used to treat CI/RI and their associated mechanisms. Figure 1 summarizes the etiopathogenesis of CI/RI, and the tables represent CHM’s probable effects and mechanism of neuroprotection activity in CI/RI models. This work opens a new window for drug discovery and brings a better understanding of the therapeutic principles of CHM for CI/RI. It is valuable for further promoting candidate drug development and providing more citation-based information that can be applied in clinical drug application against CI/RI.

## 2 Survey Methodology

“PubMed,” “ScienceDirect,” and “CNKI” databases are mainly used to search for published articles using the search terms “traditional Chinese medicines,” “Chinese herbal medicine,” “natural medicine,” “natural drug,” “crude drugs,” “cerebral,” “brain,” “ischemia,” and “stroke” in the “title/abstract” field. Related articles were chosen manually before December 31, 2020. Both animal experiments (*in vivo*) and cell studies (*in vitro*) on CHM intervention in CI/RI are included in the review.

## 3 Etiopathogenesis OF CI/RI

According to the clinical study, CI/RI arises due to blockage in the blood vessels caused by thrombus, hemorrhage in the brain. CI/RI is often induced by complicated pathologic factors, such as inflammation, oxidative stress, apoptosis, Ca^2+^ overload, autophagy, and many others. The major signaling pathways in CI/RI include PI3K/AKT, MAPKs, NF-κB, Nrf2, and others. Moreover, the numerous abovementioned factors or signaling pathways that lead to CI/RI are related to and induced with each other, eventually resulting in apoptosis or nerve necrosis in the ischemic region. The detailed process is summarized in Figure 1.

### 3.1 Inflammation

Inflammation response is a key element conducive to the pathogenic process (Dong et al., 2019; Lambertsen et al., 2019) and involved in all stages (Boehncke, 2018; O'Connell et al., 2017) of CI/RI, which is triggered by toll-like receptor 4 (TLR4) activation. TLR4, a member of the TLRs family, activates a typical transcription factor nuclear factor-kappa B (NF-κB). When cells are irritated by extracellular matters, viral products or bacterial components through TLR4 and the NF-κB nuclear localization site are exposed. Free NF-κB migrates to the nucleus at a high rate of speed, binds to specific κB sequences, and then transcribes in the nucleus (P65 or P50) after combining with promoters and enhancers of specific gene sequences (Santa-Cecilia et al., 2016; Wu et al., 2020). Once activating NF-κB p65 or NF-κB p50, inflammatory cells, such as lymphocytes, leukocytes, neutrophils, and macrophages, actively infiltrate into the brain, and then overproduced reactive oxygen species (ROS) are released. ROS promotes glial activation and neutrophil infiltration and contributes to the release of pro-inflammatory cytokines, such as tumor necrosis factor-α (TNF-α), interleukin-1β (IL-1β), IL-6, inducible nitric oxide synthase (iNOS), and nitric oxide (NO) (Kim et al., 2014; Anrather and Iadecola, 2016). After a while, some pro-inflammatory cytokines, such as TNF-α, IL-1β, and NO, exacerbate and block microvessels, further inducing the release of cytokines by activating leukocytes, and result in secondary neuronal damage and inflammation exacerbation (Ji et al., 2016; Moritz et al., 2017). Transforming growth factor-β1 (TGF-β1), IL-4, and IL-10, as anti-inflammatory cytokines, suppress the secretion of pro-inflammatory cytokines (Kim et al., 2014). If the level of anti-inflammatory cytokines is higher than the pro-inflammatory cytokine expression, the inflammation response is mitigated.

### 3.2 Oxidative Stress

Oxidative stress easily happens during CI/RI because of the more oxygen consuming; the overproduction of ROS, reactive nitrogen species (RNS), and polyunsaturated fatty acids; the fewer contents of endogenous antioxidants, and the weaker antifree radical system (Du et al., 2012). It often results from the imbalance of ROS/RNS production and antioxidant factors of cells (Kopani et al., 2006). Under normal physiological conditions, ROS can be scavenged by endogenous antioxidant enzymes, such as superoxide dismutase (SOD), catalase (CAT), and glutathione peroxidase (GPX), which are proven to combat oxidative stress (Son et al., 2010; Li et al., 2013). Nuclear factor erythroid 2-related factor (Nrf2), a pleiotropic transcription factor and the key genomic homeostatic regulator, can regulate the expression of endogenous antioxidant enzymes (Wu Q. Q. et al., 2011). However, superfluous production of ROS/RNS causes energy impairment, lipid peroxidation, mitochondrial dysfunction, accumulation of aggregated proteins, and damage of intracellular biofilm lipids, leading to oxidative stress (Olmez and Ozyurt, 2012; Navarro-Yepes et al., 2014). Oxidative stress not only leads to systemic oxidative damage, but also triggers apoptosis, pyroptosis, and inflammation.

### 3.3 Apoptosis

Apoptosis, one of the primary patterns of neuronal death, plays a critical role in pathogenesis and prognosis. TLRs activate downstream adaptor signaling molecules such as phosphatidylinositol-3-kinase-serine/threonine kinase (PI3K/AKT). Phosphorylation of PI3K/AKT can affect the B-cell leukemia/lymphoma 2 (Bcl-2) protein family, which includes the antiapoptotic protein Bcl-2. There is a proapoptotic protein called Bcl 2-associated X (Bax), which blocks programmed cell death. The Bcl-2 protein family mediates apoptosis by opening the mitochondrial permeability transition pore, thereupon bringing the release of cytochrome c (Cyt-c) from the mitochondria intermembrane space to the cytoplasm (Vela et al., 2013). Apoptosis is also caused by the activation of cysteine-aspartic proteases (Caspases), which hydrolyzes and cleaves relevant proteins and regulates cell death. In all the Caspases, Caspase-9, and Caspase-3 expression are often found in the CI rat model (Geng et al., 2013). Caspase-3, the effector/executioner compound, the cleavage of which leads to DNA fragmentation, degradation, and cross-linking of related proteins, then induces cell apoptosis directly. Caspase-9, the initiator molecule formed with Cyt-c, activates Caspase-3 and gives rise to irreversible cell death (Chen et al., 2015).

### 3.4 Ca^2+^ Overload

Ca^2+^ overload is supposed to be one of the first events induced by CI/RI. During CI/RI, the content of adenosine triphosphate (ATP) sharply drops, the cellular function is beyond paired due to energy loss, and subsequent injury in the brain is hard to restore. Under these circumstances, abundant extracellular Ca^2+^ outflow through Ca^2+^ channels into cells is called “Ca^2+^ overload” or “Ca^2+^ toxicity.” Overloaded Ca^2+^ combines with death signals to facilitate procedure change of cellular components and death by apoptosis or necrosis or straightly acts on catabolic enzymes that cause cell demise (Orrenius et al., 2003).

Transient receptor potential vanilloid 4 (TRPV4), a member of the TRP family, is widely distributed in neurons, smooth muscle cells, and glial cells. The activation of the TRPV4 channel accelerates the opening of intermediate conductance Kca (IK_Ca_), small conductance Kca (SK_Ca_), and large conductance Ca^2+^-activated K^+^ (BK_Ca_) channels and reduces the accumulation of a potentially pathological level of Ca^2+^ from a number of potential sources of influx to prevent Ca^2+^ overload (Sonkusare et al., 2012; Szarka et al., 2018). The intracellular Ca^2+^ overload brings out all kinds of Ca^2+^-dependent physiological reactions, such as the disintegration of phospholipids, proteins, and nucleic acids and the disruption of the structure and function of the cell membrane, accelerating cell death finally (Berliocchi et al., 2005).

### 3.5 Autophagy

Autophagy, a stylized cell survival process, has caught many researchers’ eyes as a novel effect of CI/RI (Hou et al., 2019). Moderate autophagy maintains cell metabolism and homeostasis to protect nerve cells (Wang P et al., 2018). If the autophagy is activated excessively, it leads to cell death and aggravation of CI/RI. The cellular events that occur during autophagy follow distinct stages: autophagy initiation, autophagosomal membrane nucleation, the fusion of the autophagosome with the lysosome (Wang M et al., 2018). The mitogen-activated protein kinase (MAPK) and PI3K/AKT pathway are responsible for the induction of autophagy. The MAPK family is composed of c-Jun N-terminal protein kinases (JNK), p38, and extracellular signal-regulated kinases (ERK). The phosphorylation of JNK and p38, the activation PI3K/AKT signal pathway, and the suppression of ERK, can motivate the mTOR signaling and then lead to autophagy initiation (Kyriakis and Avruch 2012). Beclin 1 takes part in the nucleation of the autophagosomal membrane and becomes the marker of final autophagosome formation. The microtubule-associated protein 1 light chain 3 (LC3) is the first kind of protein identified from the autophagosome membrane. During the autophagy process, LC3-I is hydrolyzed into LC3-II. Therefore, the expression of beclin 1 or LC3-II/LC3-I ratio is considered as the major criterion for judging the autophagy level (Mizushima, 2010; Mizushima and Komatsu, 2011). The final stage is the fusion of autophagosome and lysosome. The autophagy process may be conducive to neural death in a way. There is a report showing that inhibition of autophagy can block the cathepsins tBid-mitochondrial apoptotic signaling pathway via stabilization of the lysosomal membrane in ischemic astrocytes in the model of middle cerebral artery occlusion (MCAO) rats and oxygen glucose deprivation (OGD) cells (Zhou X. Y. et al., 2017).

### 3.6 Ferroptosis

Ferroptosis, a newly programmed type of regulated cell death, is the focus and hot spot of research on the treatment and prognosis improvement of many diseases in recent years (Lei et al., 2019; Qiu et al., 2020). The two key features of the ferroptosis process are the accumulation of lipid peroxidates and the metabolism of iron ions. The solute carrier family 7A11 (SLC7A11), a subunit of system Xc-, is a part of an important antioxidant system in cells. The downregulation of SLC7A11 causes suppression of glutathione (GSH) biosynthesis and the subsequent inhibition of GPX4 activity, which leads to the accumulation of lipid ROS and the activation of ferroptosis (Li J. et al., 2020). Furthermore, the deposition of iron brings ferroptosis through producing ROS by the Fenton reaction. The transferrin receptor 1 (TFR1) transports the ferric iron (Fe^3+^) into the endosome, and then Fe^3+^ converses into the ferrous iron (Fe^2+^). After that, the divalent metal transporter 1 (DMT1) mediates Fe^2+^ to release from the endosome and then stores it in the unstable iron pool and ferritin (Xie et al., 2016). The ferroportin oxidizes Fe^2+^ to Fe^3+^, thereby strictly controlling iron homeostasis and inhibiting ferroptosis in cells. It follows that TFR1 and DMT1 are vital proteins to regulate intracellular iron transportation and maintain iron metabolism and homeostasis.

### 3.7 Parthanatos

Parthanatos, a new type of caspase-independent pathophysiology, is found to play an important role during CI/RI lately (Zhang R. et al., 2015; Wang et al., 2016). In parthanatos, mitochondria homeostasis is considered to be the crucial element of cell death (Dawson and Dawson, 2017). The hexokinase-II (HK-II), the isoform of HK, has the N-terminal hydrophobic segment, which binds to mitochondria and is activated by the PI3K/AKT pathway. The poly ADP-ribose polymerase-1 (PARP-1) near the DNA damage site has the ability to repair DNA. The activation of PARP1 and HK-II trigger mitochondrial membrane depolarization and induce apoptosis inducing factor (AIF) released from mitochondria to the nucleus, which then results in DNA fragmentation, dysfunction of mitochondria, and collapse of cells (Wang et al., 2011; Nederlof et al., 2014; Robinson et al., 2019).

### 3.8 Pyroptosis

Pyroptosis, a novel and special form of programmed cell death, brings an inflammation response and aggravates damage (Zeng et al., 2019). Activating the NOD-like receptor protein 3 (NLRP3) inflammasome, which controls maturation and secretion of pro-inflammatory cytokines, is regarded as a crucial pathway in the initiation of pyroptosis (Liu Z. et al., 2017). NLRP3 is activated by an external stimulus, such as oxidative stress, and triggers the Caspase-1 cascade (Barrington et al., 2017). Caspase-1, an essential precursor of pyroptosis, cleaves gasdermin D (GSDMD) into an N-terminal product (GSDMD-NT) and C-terminal fragment (GSDMD-CT) (Shi et al., 2015; Kovacs and Miao, 2017). GSDMD-NT binds to the components of the lipid bilayer, including phosphatidic acid, phosphatidylinositol, and phosphatidylserine, leading to pore generation to execute cell swelling and rupture (Zeng et al., 2019). GSDMD-CT is different from GSDMD-NT; it suppresses the GSDMD-NT when the cell is in a quiescent condition to remain GSDMD in an inactivated status. On the other side, the Caspase-1-processed pro-inflammatory cytokines, such as IL-1β, IL-6, and IL-18, are released through membrane pores formed by GSDMD-NT. This cascade leads to pyroptosis, amplifies the inflammation and then aggravates the brain injury.

### 3.9 Other Etiologies

CI/RI triggers a complex cascade of pathophysiological events. Except for the etiologies reviewed above, there are a great many pathological mechanisms that participate in the process of CI/RI, such as BBB permeability, cerebral edema, and angiogenesis. Aquaporin 4 (AQP4), the abundant and important aquaporin, regulates Connexin 43 (Cx43) expression. Matrix metalloprotein 9 (MMP-9), a member of the family of zinc-dependent proteolytic enzymes, degrades almost all the extracellular matrix and basement membrane components. The expression of AQP4 and MMP-9 could severely affect the permeability of the BBB and the degree of cerebral edema (Yao et al., 2015; Maugeri et al., 2016). The tight junction (TJ), a layered cell structure construct, plays a crucial role in the integrity of the BBB. The expression of TJ-associated proteins, such as claudin-5, occluding, and zona occludens-1 (ZO-1), is closely related to BBB permeability (Yang and Rosenberg, 2011). Angiogenesis, including the cell proliferation, migration, and differentiation process, is regulated by many factors such as angiogenin (Ang), vascular endothelial growth factor (VEGF), hypoxia inducible factor (HIF), and transforming growth factor (TGF). Ang-2 facilitates angiogenesis, whereas Ang-1 accelerates the subsistence of endothelial cells and the maturation of blood vessels (Anisimov et al., 2013). TGF-β1 induces VEGF to offer a favorable condition for angiogenesis through adjusting the expression of multifarious angiogenic molecules. HIF-1 increases levels of several angiogenic genes to promote forming new blood vessels (Pereira et al., 2014).

## 4 CHM for Treating CI

### 4.1 An Overview of Relevant CHM

**FIGURE 2:**
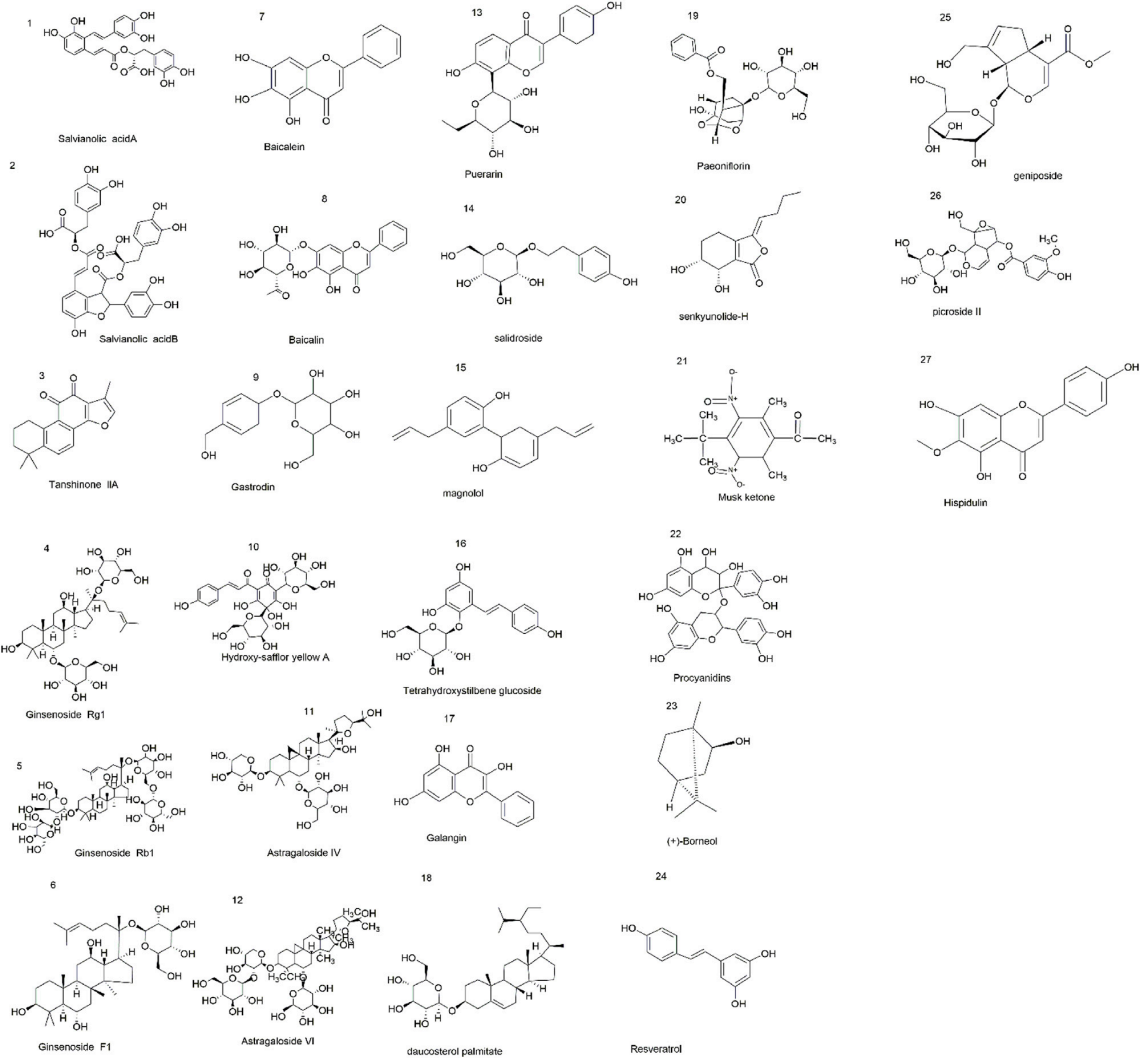
Chemical structures of extraction components with neuroprotective effects in CI/RI.

CHM is derived from natural sources and was used clinically in human history. Now, many studies suggest that CHM has many positive effects on the brain, and these effects are beneficial to the treatment and prevention of CI/RI. CHM plays an essential role in the discovery and development of new drug entities, for example, the remarkable contribution of artemisinin in combating malaria, which is threatening millions of lives in developing regions. CHM includes effective extraction components of single CHM and CHM formulas and preparations. Researchers find that some CHM extractions, such as salvianolic acid B (Jiang et al., 2015; Wang G. et al., 2019), salvianolic acid A (Wang et al., 2012), tanshinone II_A_ (Cai et al., 2017; Xu, 2019), ginsenoside Rg1 (Zhang et al., 2013; He et al., 2014; Sun et al., 2014), ginsenoside Rb1 (Zhou Y. et al., 2014; Chen et al., 2015), ginsenoside F1 (Zhang J. et al., 2019), baicalein (Li W. H. et al., 2020), baicalin (Zhou et al., 2016; Zhou Z. Q. et al., 2017), gastrodin (Peng et al., 2015; Liu et al., 2016; Shi et al., 2018), hydroxy-safflor yellow A (Tan et al., 2020), astragaloside IV (Li et al., 2019), astragaloside VI (Chen X et al., 2019), puerarin (Zhou F. et al., 2014; Liu Y. et al., 2017), salidroside (Han et al., 2015), magnolol (Liu X. et al., 2017), tetrahydroxystilbene glucoside (Yu et al., 2019), galangin (Guan et al., 2021), daucosterol palmitate (Zhang H. et al., 2020), senkyunolide-H (Zhang J. Y. et al., 2019), paeoniflorin (Guo et al., 2012; Chen et al., 2013), musk ketone (Zhou et al., 2020), procyanidins (Yang et al., 2020), ilexonin A (Zheng et al., 2015; Xu et al., 2016), picroside II (Zhang et al., 2017), geniposide (Li et al., 2016), resveratrol (Sun et al., 2019; Zhang, 2019), hispidulin (An et al., 2019), and (+)-borneol (Chang et al., 2017), have a potent neuroprotection effect against CI/RI. Here, Figure 2 represents chemical structures related to compounds from single CHM that improves CI/RI. As we all know, in clinical treatment, the application of CHM formulas/preparations is widespread, and most of them are confirmed to have a good therapeutic effect against CI/RI. CHM protects CI/RI from many aspects; it ameliorates oxidative stress, inflammation, and neuron apoptosis; modulates intracellular Ca^2+^ concentration, ferroptosis, and parthanatos; promotes angiogenesis; and regulates autophagy and pyroptosis. The related intracellular signaling pathways are also intricate. In a word, exploring the mechanism and effective molecular targets of CHM are important and will provide new insights into the treatment of CI/RI and promote the process of discovering active drugs from CHM.

Traditional Chinese medicine theory believes that blood stasis is the fundamental cause of CI/RI. CHM plays an irreplaceable role and provides unique advantages in the management of CI/RI. Some scholars identify that CHM has actions of activating Qi and blood, clearing heat and detoxification, nourishing Yin and generating body fluid, and calming liver wind in treating and preventing CI (Wang, 2016). Increasing evidence indicates CHM might be effective in the treatment of CI/RI, especially CHM with the effects of replenishing and activating blood. Extraction components of single CHM (Table 1), CHM formulas (Table 2), and CHM preparations (Table 3) all show some benefit in removing blood stasis, which drives further studies for ischemic diseases more widely.

**TABLE 1:** Recent advances in protective mechanisms of extraction components of single CHM for CI/RI.

Single botanical drugs (main active ingredients)	Object	Effect/Mechanism	Controls	Minimal active dosage	References
*Salvia miltiorrhiza* Bunge [Lamiaceae; *Salviae miltiorrhizae* radix et rhizoma] (SAA)	MCAO/R-induced rats (*in vivo*)	inhibits soluble epoxide hydrolase (sEH) to increase epoxyeicosatrienoic acids (EETs) levels	sham-operated group (negative control); 14,15-EEZE group (positive control)	0.3 mg/kg (i.v.)	Wang et al. (2012)
*Salvia miltiorrhiza* Bunge [Lamiaceae; *Salviae miltiorrhizae* radix et rhizoma] (SAB)	MCAO/R-induced mice/rats (*in vivo*)	increases the activities of SOD, CAT, GSH-Px and Bcl-2, decreases the content of MDA, LDH, NOS and Bax	sham-operated group (negative control); nimodipine group (positive control)	12 mg/kg (i.p.)	Jiang et al. (2015); Wang G. et al. (2019)
*Salvia miltiorrhiza* Bunge [Lamiaceae; *Salviae miltiorrhizae* radix et rhizoma] (tanshinone IIA)	OGD/R-induced HT22 cells (*in vitro*); MCAO/R-induced mice (*in vivo*)	inhibits ferroptosis by regulating the iron homeostasis, reducing the intracellular ROS level and the iron deposition; induces oxidation by upregulating the expression of Nrf2	erastin group (negative control); Nrf2 knockout mice group (negative control)	25 mg/kg (i.p.); 1 μmol/L (incubate)	Xu (2019); Cai et al. (2017)
*Panax ginseng* C.A.Mey. (Araliaceae; *Ginseng* radix et rhizoma) (GRg1)	MCAO/R-induced mice/rats (*in vivo*); OGD/R-induced neuronal cells (*in vitro*)	prevents neurotoxicity by blocking Ca^2+^ over-influx into neuronal cells to elevate intracellular Ca^2+^ concentration	sham-operated group (negative control); acetazolamide group (positive control)	20 mg/kg (i.p.); 5 μmol/L (incubate)	He et al. (2014); Zhang et al. (2013); Sun et al. (2014)
*Salvia miltiorrhiza* Bunge [Lamiaceae; *Salviae miltiorrhizae* radix et rhizoma] (GRb1)	2-VO/MCAO/R-induced mice/rats (*in vivo*); OGD/R-induced neuronal cells (*in vitro*)	Protects CI/IR by antioxidant, anti-inflammatory, and antiapoptotic activities and involve the inhibition of excitotoxicity and Ca^2+^ influx, preservation of blood–brain barrier (BBB) integrity, and maintenance of energy metabolism	sham-operated group (negative control); nimodipine group (positive control); LY294002 group (positive control)	5 mg/kg (i.p.); 1 μmol/L (incubate)	Xie et al. (2021)
*Panax ginseng* C.A.Mey.(Araliaceae; *Ginseng* radix et rhizoma) (GF1)	OGD/R-induced umbilical vein endothelial cells (UVECs); brain microvascular endothelial cells (BMECs) (*in vitro*)	promotes angiogenesis by IGF-1/IGF1R signaling pathway	VEGF group (positive control)	20 μmol/L (incubate)	Zhang J et al., (2019)
*Scutellaria baicalensis* Georgi [Lamiaceae; *Scutellariae* radix] (baicalein)	MCAO/R-induced rats (*in vivo*)	attenuates parthanatos by inhibiting the activation of PARP-1 and diminishing the release of AIF	sham-operated group (negative control)	100 mg/kg (i.p.)	Li W. H et al., 2020
*Scutellaria baicalensis* Georgi [Lamiaceae; *Scutellariae* radix] (baicalin)	HIE-induced rats (*in vivo*); OGD/R-induced SH-SY5Y cells (*in vitro*)	suppresses apoptosis by regulating NF-κB and PI3K/Akt signaling pathways	sham-operated group (negative control); LY294002 group (positive control)	1 μmol/L (incubate); 120 mg/kg (i.p.)	Zhou Z. Q et al. (2017); Zhou et al. (2016)
*Gastrodia elata* Blume [Orchidaceae; *Gastrodiae* rhizome] (GAS)	MCAO/R-induced rats (*in vivo*); H_2_O_2_/R-induced cells (*in vitro*)	improves antioxidant, anti-inflammation and antiapoptotic activities by activating Akt/Nrf2 pathway; suppressing expression of Caspase-3, Bax, TNF-α, IL-1β, and MDA; upregulating amount of SOD, HO1, SOD1, and Bcl-2 content	sham-operated group (negative control); nimodipine group (positive control)	50 mg/kg (i.p.); 15 μg/ml (incubate)	Peng et al. (2015); Liu et al. (2016); Shi et al. (2018)
*Carthamus tinctorius* L. (CTL) [Asteraceae; *Carthami* Flos] (HSYA)	MCAO/R-induced rats (*in vivo*); H_2_O_2_/R-induced cells (*in vitro*)	inhibits pyroptosis by regulating NF-κB signaling pathway and the levels of NLRP3 and Caspase-1; improves anti-apoptotic by activating PI3K/Akt signaling pathway	sham-operated group (negative control); lexiscan group (positive control)	10 mg/kg (i.v.); 10 μg/ml (incubate)	Tan et al. (2020)
*Astragalus mongholicus* Bunge [Fabaceae; *Astragali* radix] (AIV)	MCAO/R-induced rats (*in vivo*); OGD/R-induced neuronal cells/SH-SY5Y cells (*in vitro*)	reduces apoptosis and parthanatos by activating AKT to promote HK-II binding to mitochondria; improves antioxidant and antiapoptosis by regulating JAK2/STAT3 signaling pathway	sham-operated group (negative control); triciribine group and AG490 group (positive control)	15 mg/kg (i.v.); 10 μg/ml (incubate)	Li et al. (2019); Xu et al. (2020)
*Astragalus mongholicus* Bunge [Fabaceae; *Astragali* radix] (AVI)	MCAO/R-induced rats (*in vivo*); cultured neural stem cells (*in vitro*)	promotes the proliferation and neurogenesis of cells, and repairs neurological function of rats by activating EGFR/MAPK signaling pathway	sham-operated group (negative control); gefitinib/PD98059 group (negative control)	2 μg/kg (i.v.); 10 μg/ml (incubate)	Chen X et al., 2019
*Pueraria montana* var. *lobata* (Willd.) Maesen and S.M.Almeida ex Sanjappa & Predeep [Fabaceae; *Puerariae lobatae* radix] (puerarin)	MCAO/R-induced rats (*in vivo*); OGD/R-induced brain vascular endothelial cells (BVECs) (*in vitro*)	reduces infarct size and brain water content by regulating TLR4/NF-κB signaling pathway; reduces apoptosis by regulating ERK and PI3K/AKT/mTOR signaling pathways	sham-operated group (negative control)	100 mg/kg (i.p.); 10 μg/ml (incubate)	Zhou F et al. (2014); Liu Z et al. (2017)
*Rhodiola crenulata* (Hook.f. and Thomson) H.Ohba [Crassulaceae; *Rhodiolae crenulatae* radix et rhizome](salidroside)	MCAO/R-induced rats (*in vivo*)	reduces oxidative stress by regulating Nrf2 signaling pathway	sham-operated group (negative control)	15 mg/kg (i.p.)	Han et al. (2015)
*Magnolia officinalis* Rehder and E.H.Wilson [Magnoliaceae; *Magnoliae officinalis* cortex] (magnolol)	MCAO/R-induced mice (*in vivo*); OGD/R-injured BMECs (*in vitro*)	suppresses inflammation by inhibiting TNF-α, IL-1β, and NO expressions; improves BBB function by increasing ZO-1 and occludin levels	sham-operated group (negative control); edaravone group (positive control)	1.4 μg/kg (i.v.); 1 μmol/L (incubate)	Liu X et al., 2017
*Reynoutria multiflora* (Thunb.) Moldenke [Polygonaceae; *Polygoni multiflora* radix] (tetrahydroxystilbene glucoside)	MCAO/R-induced mice (*in vivo*)	mitigates apoptosis and autophagy by elevating the expressions of NOX4, caspase-3/-9, Beclin 1, and the LC3BII/I ratio	sham-operated group (negative control)	3 mg/kg (i.v.)	Yu et al. (2019)
*Alpinia officinarum* Hance [Zingiberaceae; *Alpiniae officinarum* rhizoma] (galangin)	MCAO/R-induced gerbils (*in vivo*)	inhibits ferroptosis by enhancing the expressions of SLC7A11 and GPX4	sham-operated group (negative control)	25 mg/kg (i.p.)	Guan et al. (2021)
*Alpinia oxyphylla* Miq. [Zingiberaceae; *Alpiniae oxyphyllae* fructus] (daucosterol palmitate)	MCAO/R-induced rats (*in vivo*)	inhibits apoptosis by regulating PI3K/Akt/mTOR signaling pathway	sham-operated group (negative control); wortmannin group (positive control)	20 mg/kg (i.p.)	Zhang H et al., 2020
*Conioselinum anthriscoides* ‘Chuanxiong’ [Apiaceae; *Chuanxiong* rhizoma] (senkyunolide-H)	MCAO/R-induced mice (*in vivo*); OGD/R-PC12 cells (*in vitro*)	inhibits inflammation and apoptosis by activating PI3K/Akt/NF-κB signaling pathway	sham-operated group (negative control)	20 mg/kg (i.g.)	Zhang J. Y et al., 2019
*Paeonia lactiflora* Pall. [Paeoniaceae; *Paeoniae* radix rubra] (paeoniflorin)	MCAO/R-induced mice/rats (*in vivo*); PDGF-stimulated VSMC/A7r5 cells (*in vitro*)	ameliorates CI and arterial intimal hyperplasia by modulating Ras/MEK/ERK signaling pathway; reduces inflammation by inhibiting MAPKs/NF-κB signaling pathway	sham-operated group (negative control)	0.5 mg/kg (i.g.); 5 mg/kg (i.p.); 5 μmol/L (incubate)	Chen et al. (2013); Guo et al. (2012)
*Cucumis melo* L. [Cucurbitaceae; Trichosanthis fructus] (musk ketone)	MCAO/R-induced rats (*in vivo*); OGD/R-induced Neural stem cells (NSCs) (*in vitro*)	induces NSC proliferation and differentiation by activating the PI3K/Akt signaling pathway	sham-operated group (negative control)	0.5 mg/kg (i.p.)	Zhou et al. (2020)
grape seeds (procyanidins)	MCAO/R-induced rat (*in vivo*); OGD/R-BV2 cells (*in vitro*)	exerts neuroprotective effect by inhibiting TLR4-NLRP3 inflammasome signaling pathway	sham-operated group (negative control)	20 mg/kg (i.g.)	Yang et al. (2020)
*Ilex pubescens* Hook. and Arn. [Aquifoliaceae; *Ilex pubescens* radix] (ilexonin A)	MCAO/R-induced rats (*in vivo*); OGD/R- Neural stem cells (NSCs) (*in vitro*)	promotes revascularization, neuronal regeneration, and regulates astrocyte and microglia cell activation; promotes neuronal proliferation and regeneration by regulating Wnt signaling pathway	sham-operated group (negative control)	20 mg/kg (i.p.)	Xu et al. (2016); Zheng et al. (2015)
*Picrorhiza kurroa* Royle ex Benth. [Plantaginaceae; *Picrorhizae* rhizoma] (picroside II)	MCAO/R-induced rats (*in vivo*)	inhibits apoptosis and inflammation by scavenging ROS content and regulating Cyt-c expression	sham-operated group (negative control); CyclosporinA group (positive control)	20 mg/kg (i.p.)	Zhang et al. (2017)
*Gardenia jasminoides* J.Ellis [Rubiaceae; *Gardeniae* fructus] (geniposide)	OGD/R-induced BMECs (*in vitro*)	attenuates inflammation by suppressing ERK1/2 signaling pathway	sham-operated group (negative control)	33.2 μg/ml (incubate)	Li et al. (2016)
*Polygonum cuspidatum* Sieb. et Zucc. [Polygonaceae; *Polygoni cuspidate* radix et rhizoma] (resveratrol)	MCAO/R-induced rats (*in vivo*); H_2_O_2_/R-induced RSC96 cells (*in vitro*)	inhibits pyroptosis and autophagy by blocking the activation of NLRP3 inflammasome, Caspase-1, mTOR phosphorylation	sham-operated group (negative control)	0.1 mmol/L (incubate)	Sun et al. (2019); Zhang (2019)
*Saussurea involucrata* (Kar. and Kir.) Sch.Bip. [Asteraceae; *Saussureae involucratae* flos] (hispidulin)	MCAO/R-induced rats (*in vivo*); OGD/R-induced astrocytes (*in vitro*)	suppresses NLRP3-mediated pyroptosis by modulating the AMPK/GSK3β signaling pathway	sham-operated group (negative control)	40 mg/kg (i.p); 5 μmol/L (incubate)	An et al. (2019)
*Blumea balsamifera* (L.) DC. [Asteraceae; *Blumi balsamiferae* herba] ((+)-borneol)	MCAO/R-induced rats (*in vivo*); lipopolysaccharide (LPS)-induced BV2 microglial cells (*in vitro*)	suppresses inflammation by reducing production of proinflammatory cytokines (iNOS and TNF-α)	sham-operated group (negative control)	0.5 mg/kg (i.v.)	Chang et al. (2017)
*Ginkgo biloba* L. [Ginkgoaceae; *Ginkgo folium*] (ginkgo diterpene lactones)	MCAO/R-induced rats (*in vivo*); OGD/R-induced cortical astrocytes (*in vitro*)	suppresses inflammation by regulating TLR4/NF-κB signaling pathway	sham-operated group (negative control)	1.25 mg/kg (i.v.); 12.5 μg/ml (incubate)	Li X. et al., 2020
*Dendrobium nobile* Lindl. [Orchidaceae; *Dendrobii* caulis] (dendrobium alkaloids)	MCAO/R-induced rats (*in vivo*); OGD/R-induced HT22 cells (*in vitro*)	inhibits pyroptosis by reducing the levels of Caspase-1, gasdermin-D and inflammatory factors (IL-1β, IL-6, and IL-18)	sham-operated group (negative control); belnacasan group (positive control)	0.5 mg/kg (i.p.); 0.03 mg/ml (incubate)	Liu D. et al., 2020
*Ilex pubescens* Hook. and Arn. [Aquifoliaceae; *Ilex pubescens* radix] total flavonoids	MCAO/R-induced rats (*in vivo*)	suppresses inflammation by decreasing proinflammatory cytokines (NO, IL-1β, TNF-α, TNOS, iNOS, and cNOS) and increasing anti-inflammatory cytokine (IL-10); suppresses oxidative stress by decreasing (MDA) level and increasing SOD content	sham-operated group (negative control)	100 mg/kg (i.g.)	Fang et al. (2017); Miao et al. (2016)
*Achyranthes bidentata* Blume [Amaranthaceae; *Achyranthis bidentatae* radix] (Achyranthes bidentata polypeptide k)	MCAO/R-induced rats (*in vivo*); OGD/R-induced rat endothelial cells (*in vitro*)	improves recognition abilities and neurological outcomes by modulating NF-κB and MMP-2/-9	sham-operated group (negative control)	0.1 mg/kg (i.v.); 0.2 μg/ml (incubate)	Cheng et al. (2019)
*Rhododendron tomentosum* Harmaja [Ericaceae; *Rhododendri* flos] total favonoids	MCAO/R-induced rats (*in vivo*)	reduces Ca^2+^ concentration in the cell by activating BKCa channel through a TRPV4-dependent signaling pathway	sham-operated group (negative control)	100 mg/kg (i.v.)	Han et al. (2018)
*Cordyceps sinensis* (Berk. Sacc.) [Clavicipitaceae; *Cordyceps*] extract	MCAO/R-induced rats (*in vivo*); OGD/R-induced BMECs (*in vitro*)	inhibits apoptosis by regulating the expressions of Bax/Bcl-2, Cyt-c, and caspase-3/-8/-9	sham-operated group (negative control)	1 g/kg (i.g.); 5 μg/ml (incubate)	Bai et al. (2020)
*Pinellia pedatisecta* Schott [Araceae; *Pinellia* rhizoma] extract	MCAO/R-induced rats (*in vivo*); OGD/R-induced UVECs (*in vitro*); OGD/R-induced BMECs (*in vitro*)	inhibits apoptosis by keeping the balance between Bcl-2 and Bax	sham-operated group (negative control); nimodipine group (positive control)	5 mg/kg (i.g.)	Ye et al. (2016)

**TABLE 2:** Recent advances in protective mechanisms of CHM formulations for CI/RI.

CHM formulations	Object	Effect/Mechanism	Controls	Minimal active dosage	References
BHT	MCAO/R-induced rats (*in vivo*); H_2_O_2_/R-induced HUVECs (*in vitro*)	inhibits inflammation by regulating decreasing proinflammatory cytokines (IL-6, TNF-α) and increasing anti-inflammatory cytokine (IL-10); maintains the BBB integrity by inhibiting the activation of the HIF-1 α/VEGF pathway and stabilizing ion channel of β-ENaC in brain; promotes angiogenesis by modulating SIRT1/VEGF and Nox4/ROS signaling pathways	sham-operated (negative control)	0.5 g/kg (i.g.); 10 mg/ml (incubate)	Chen K. Y. et al. (2020); Chen Z. Z et al. (2019); Zheng et al. (2018); Shen et al. (2020)
HJT	MCAO/R-induced rats (*in vivo*); OGD/R-induced cerebral cortical neuron (*in vitro*)	inhibits apoptosis by activating the PI3K/AKT signaling pathway and HIF-1α; induces protective autophagy by regulating MAPK-mTOR signaling pathway	sham-operated (negative control)	2.5 g/kg (i.g.); 0.1 mg/ml (incubate)	Zhang Q. et al. (2014); Wang et al. (2013)
TST	MCAO/R-induced rats (*in vivo*)	inhibits inflammation, apoptosis by regulating the expressions of HIF-1α, TNF-α, iNOS and Caspase-3; protects CI/RI by modulating the expressions of brain-derived neurotrophic factor and p53	sham-operated (negative control)	0.7 g/kg (i.g.)	Wu C. J. et al. (2011); Wu, (2018); Fan et al. (2015)
NTF	MCAO/R-induced rats (*in vivo*)	inhibits ferroptosis through adjusting the TFR1/DMT1 and SCL7A11/GPX4 signaling pathways and the expression of Ferroportin to balance iron levels	sham-operated (negative control); deferiprone (positive control)	0.7 g/kg (i.g.)	Liao et al. (2015); Lan et al. (2020)
QY	MCAO/R-induced mice (*in vivo*)	inhibits inflammation by modulating IL-1β, IL-6, TNF-α, NF-κB p65, TGF-β1, TLR4, and Interferon-γ	sham-operated (negative control); edaravone (positive control)	5.69 mg/ml (i.g.)	Wang Y. et al., 2020
SKD	MCAO/R-induced rats (*in vivo*)	mitigates apoptosis and oxidation by upregulating SOD and GSHPx levels, downregulating iNOS, TNOS, and Caspase-3 expressions	sham-operated (negative control); nimodipine (positive control)	0.7 g/kg (i.g.)	Chen et al. (2014)
GGD	MCAO/R-induced rats (*in vivo*)	mitigates oxidation by regulating SOD, MDA and GSHPx levels; inhibits inflammation by modulating NF-κB signaling pathway; inhibits ferroptosis by regulating Poly (ADP-ribose) (PAR) polymerase-1 (PARP-1)/apoptosis-inducing factor (AIF) signaling pathway; mitigates intracellular Ca^2+^ overload by regulating the concentration of Ca^2+^	sham-operated (negative control)	3.6 g/kg (i.g.)	Zhang S. et al. (2015); Hu et al. (2015); Nan et al. (2020); Hu et al. (2018)
MYF	MCAO/R-induced rats (*in vivo*)	inhibits ferroptosis by regulating the expressions of Bcl-2/Bax, Cyt-c, Caspase-9/-3/-7; mitigates oxidation by modulating the content of SOD, LDH, CAT, MDA, and GSH-PX; inhibits autophagy by activating AMPK/mTOR signaling pathway	sham-operated (negative control); nimodipine (positive control)	58 mg/kg (i.g.)	Zhao et al. (2016); Wang et al. (2017); Chen et al. (2016)
STL	MCAO/R-induced rats (*in vivo*)	exerts anti-inflammation and antiapoptosis effects through activating the SIRT1 signaling pathway	sham-operated (negative control)	5.7 ml/kg (i.g.)	Mei et al. (2017)

**TABLE 3:** Recent advances in protective mechanisms of CHM preparations for CI/RI.

CHM preparations	Object	Effect/Mechanism	Controls	Minimal active dosage	References
ANW	MCAO/R-induced rats (*in vivo*)	protects BBB integrity by inhibiting the activations of MMP-2 and MMP-9 and upregulating the expressions of ZO-1 and claudin-5	sham-operated group (negative control)	257 mg/kg (i.g.)	Tsoi et al. (2019a); Tsoi et al. (2019b)
QLI	MCAO/R-induced rats (*in vivo*)	inhibits apoptosis and inflammation by modulating the AMPK/NLRP3 signaling pathway; protects BBB integrity by regulating expression of TJs and HIF-1α/MMP-9	sham-operated group (negative control)	3 ml/kg (i.p.)	Ma et al. (2019); Zhang S. et al. (2020)
TLC	MCAO/R-induced rats (*in vivo*); Human cardiac microvascular endothelial cells (HCMECs) (*in vitro*)	inhibits apoptosis by regulating the PI3K/Akt signaling pathway and Connexin 43/Calpain II/Bax/Caspase-3 signaling pathway; enhances neurogenesis and angiogenesis; inhibits inflammation by downregulating AQP4, TLR4, NF-κB, and TNF-α expressions	sham-operated group (negative control); nimodipine (positive control)	0.5 g/kg (i.g.); 200 μg/ml (incubate)	Yu et al. (2019); Cheng et al. (2017); Li et al. (2015); Cai et al. (2016)
DHI	MCAO/R-induced rats (*in vivo*); H2O2/R-induced mouse Neuro-2A cells (*in vitro*)	inhibits apoptosis by regulating PI3K/Akt signaling pathway; prevents brain damage by activating Nrf2/ARE signaling pathway; inhibits inflammation by regulating NF-κB signaling pathway; improves the relative mitochondrial reductase activity of the cultured neurons	sham-operated group (negative control); edaravone/ginaton group (positive control)	0.72 ml/kg (i.p.)	Feng et al. (2020); Guo, H. et al. (2014); Jiang and Lian et al. (2015); Orgah J. Q. et al. (2019)
PTH	MCAO/R-induced rats (*in vivo*)	inhibits apoptosis and inflammation by regualting Cyt-c, Bax, P53, Caspase-3/-9 Bcl-xl, AKT, and GSK-3β	sham-operated group (negative control)	257 mg/kg (i.g.)	Zhang X. et al., 2018
ZFG	MCAO/R-induced rats (*in vivo*); OGD/R-induced BMECs (*in vitro*)	protects angiogenesis via Notch and Wnt signaling pathways	sham-operated group (negative control)	10 ml/kg (i.g.)	Huang et al. (2019)
HHS	MCAO/R-induced rats (*in vivo*); OGD/R-induced HUVECs (*in vitro*)	promotes angiogenesis by regulating HIF-1α/VEGF and stromal cell derived factor-1 (SDF-1)/cxc chemokine receptor 4 (CXCR4) signaling pathways; improves neurological function and survival by activating BDNF/PI3K/Akt signaling pathways	sham-operated group (negative control) and ginaton group (positive control)	5.1 g/kg (i.g.)	Xiang et al. (2019); Chang et al. (2016); Zhang Q. et al. (2012)
YFPI	MCAO/R-induced mice (*in vivo*); OGD/R-induced bEnd.3 cells (*in vitro*)	improves BBB dysfunction by increasing expressions of tight junction proteins and inhibiting the NF-κB signaling pathway	sham-operated group (negative control) and XueShuanTong Injection group (positive control)	336 mg/kg (i.p.); 100 μg/ml (incubate)	Cao et al. (2016); Pan et al. (2020)
SMI	MCAO/R-induced rats (*in vivo*)	attenuates autophagy by modulating the AMPK, mTOR, and JNK pathways; maintains BBB integrity by regulating the TJ-associated proteins	sham-operated group (negative control); 3-meth-yladenine group (positive control)	1.42 g/kg (i.p.)	Yang et al. (2016); Xu (2019)
XSTI	MCAO/R-induced rats (*in vivo*); OGD/R-induced SH-SY5Y cells (*in vitro*)	exerts long-term neuroprotection by inhibiting the ROCKII pathway	sham-operated group (negative control)	20 mg/kg (i.v.)	Zhou et al. (2021)

### 4.2 Extraction Components of Single CHM for Treating CI/RI

#### 4.2.1 Salvia miltiorrhiza Bunge [Lamiaceae; Salviae miltiorrhizae radix et rhizoma].

*Salvia miltiorrhiza* Bunge [Lamiaceae; *Salviae miltiorrhizae* radix et rhizoma] is one of the typical blood-activating and stasis-resolving medicines, and it offers therapeutic promise for cerebrovascular diseases (Ma Y. et al., 2020). Salvianolic acids and tanshinones are the main compounds in *Salvia miltiorrhiza* Bunge [Lamiaceae; *Salviae miltiorrhizae* radix et rhizoma]. Salvianolic acid A (SAA) is proved to display potent cerebro-protection through inhibiting soluble epoxide hydrolase (sEH) to increase epoxyeicosatrienoic acid (EET) levels (Wang et al., 2012). Some studies find that salvianolic acid B (SAB) increased the activities of SOD, CAT, and GSH-Px; decreased the content of MDA and the activities of LDH and NOS; improved Bcl-2 expression; and reduced Bax expression to exert the neuroprotective effect in animal models with CI/RI (Jiang et al., 2015; Wang G. et al., 2019). A study conducted in the model of CI/RI mice by Xu (2019) suggests that tanshinone II_A_ inhibits ferroptosis through regulating iron homeostasis, reducing the intracellular ROS level and iron deposition. Another trial identifies that tanshinone II_A_ has an antioxidant effect against CI/RI in rats. Tanshinone II_A_ upregulated the expression of Nrf2 mRNA and the content of Nrf2 protein in nuclear and then increased the content of antioxidant enzymes and reduced the generation of oxidative productions (Cai et al., 2017).

#### 4.2.2 Panax ginseng C.A.Mey(Araliaceae; Ginseng Radix et Rhizoma)

*Panax ginseng* C.A.Mey(Araliaceae; *Ginseng* radix et rhizoma), which belongs to the Araliaceae family, has the function of strengthening vital Qi for brain protection. Ginsenosides are considered the main bioactive constituent of ginseng, and some studies report their neuroprotective effects (Ong et al., 2015). Recent research indicates that ginsenoside Rg1 (GRg1) prevents neurotoxicity by blocking Ca^2+^ over-influx into neuronal cells to elevate intracellular Ca^2+^ concentration in both MCAO/R-induced rats and OGD/R-induced neuronal cells (Zhang et al., 2013; He et al., 2014; Sun et al., 2014). Ginsenoside Rb1 (GRb1) exerts significant neuroprotective effects on CI/IR both *in vivo* and *in vitro*, and these network actions and underlying mechanisms are mediated by antioxidant, anti-inflammatory, and antiapoptotic activities and involve the inhibition of excitotoxicity and Ca^2+^ influx, preservation of BBB integrity, and maintenance of energy metabolism (Xie et al., 2021). Ginsenoside F1 (GF1) promotes angiogenesis *in vitro* and *in vivo* by activating the insulin-like growth factor 1/insulin-like growth factor 1 receptor (IGF-1/IGF1R) signaling pathway (whose axis is crucial for cerebral angiogenesis and neurogenesis) in endothelial cells. Meanwhile, it also increased the cerebral microvessel density, improved cerebral blood flow in ischemic regions, and reduced CI/RI, indicating that GF1 is a promising drug for promoting recovery from CI/RI by inducing angiogenesis (Zhang J. et al., 2019).

#### 4.2.3 Scutellaria baicalensis Georgi [Lamiaceae; Scutellariae Radix]

*Scutellaria baicalensis* Georgi [Lamiaceae; *Scutellariae* radix] is an important CHM with the functions of clearing away heat and dampness, purging fire, and detoxification (Zhao et al., 2019). Baicalein and baicalin are the main active components that are extracted from *Scutellaria baicalensis* Georgi [Lamiaceae; *Scutellariae* radix]. Li *et al.* find that baicalein treatment significantly inhibits the activation of PARP-1 and nuclear translocation of AIF, diminishes the release of AIF in CI/RI rats, and thereby attenuates parthanatos and protects the cerebral tissues from ischemic injury (Li W. H. et al., 2020). Several experiments confirm that baicalin has neuroprotective effects against CI/RI by regulating the NF-κB signaling pathway and PI3K/Akt signaling pathway *in vitro* and *in vivo* (Zhou Z. Q. et al., 2016; Zhou Z. Q. et al., 2017).

#### 4.2.4 Gastrodia elata Blume [Orchidaceae; Gastrodiae Rhizome]

*Gastrodia elata* Blume [Orchidaceae; *Gastrodiae* rhizome] is a famous CHM that has been traditionally used for the treatment and prevention of CI/RI for centuries (Liu et al., 2018). Gastrodin (GAS), a phenolic extracted from *Gastrodia elata* Blume [Orchidaceae; *Gastrodiae* rhizome], was revealed to improve antioxidant, anti-inflammatory, and antiapoptotic activities in CI/RI models. In MCAO/R-induced rats and H_2_O_2_/R-induced cells, some researchers indicate that GAS suppressed expressions of Caspase-3 and Bax; upregulated the amount of SOD and the expressions of HO-1 and SOD1; and decreased levels of Bcl-2, TNF-α, IL-1β, and MDA. Moreover, it significantly activated the Akt/Nrf2 signaling pathway (Peng et al., 2015; Liu et al., 2016; Shi et al., 2018). It is worth noting that the adverse drug reaction (ADR) or event (ADE) of GAS cannot be ignored because there is a retrospective study with ADR or ADE induced by GAS (Zheng et al., 2018). Therefore, it is necessary to work out a more detailed toxicity study of GAS according to the International Council for Harmonization safety guidelines.

#### 4.2.5 Carthamus tinctorius L (CTL) [Asteraceae; Carthami Flos]

*Carthamus tinctorius* L (CTL) [Asteraceae; *Carthami* Flos] has functions on removing blood stasis and promoting blood circulation and has been used for treating coronary heart disease and cerebral thrombosis. It ameliorates CI/RI rats with antiapoptosis, antioxidant, and anti-inflammatory effects. CTL regulates the expressions of Bcl-2, Bax, Caspase-3, and MMP-9 (Chang et al., 2020); changes the content of TNF-α and IL-1β (Fu et al., 2016); and modulates the TNF-α/MAPK (Wang et al., 2012) and JAK2/STAT3 signaling pathways (Hu et al., 2017). Hydroxy-safflor yellow A (HSYA), the active chalcone glycoside extracted from CTL, can alleviate CI/RI by inhibiting pyroptosis in a multitarget way. HSYA downregulates the levels of NLRP3 and Caspase-1, the dissociation of GSDMD, and the generation of the pore. What is more, HSYA restrains activation of the NF-κB pathway to reduce the production of pro-inflammatory factors, such as IL-1β and IL-18 (Tan et al., 2020). A study reported by Chen and colleagues reveals that HSYA protects against CI/RI by an anti-apoptotic effect through modulating the PI3K/Akt signaling pathway in rats (Chen et al., 2013).

#### 4.2.6 Astragalus mongholicus Bunge [Fabaceae; Astragali Radix]

*Astragalus mongholicus* Bunge [Fabaceae; *Astragali* radix] is recorded in “Shennong’s Classic of Materia Medica” with the effects of stagnation and diuresis, which are commonly used in clinical trials of CHM. Astragaloside IV (AIV) and astragaloside VI (AVI) are natural saponins that are abundant in *Astragalus mongholicus* Bunge [Fabaceae; *Astragali* radix], which have neuroprotective effects against CI/RI through reducing oxidative stress, apoptosis, and inflammation (Jia et al., 2014; Wang et al., 2017). In a study by Li et al. (2019), to investigate the parthanatos in CI/RI, the author shows that AIV activates AKT to promote HK-II binding to mitochondria and changes mitochondria’s structure. The structural and functional integrity of mitochondria is essential for AIV to protect neuronal survival from brain damage. There is a study reporting that AIV also alleviates CI/RI by activating the Janus kinase 2 and signal transducer and activator of transcription 3 (JAK2/STAT3) signaling pathway (Xu et al., 2020). AVI activated the EGFR/MAPK signaling pathway to promote the proliferation and neurogenesis of neural stem cells and further to improve the repair of neurological function in the MCAO/R model of rats (Chen X et al., 2019).

#### 4.2.7 Pueraria montana var. Lobata (Willd.) Maesen and S.M.Almeida Ex Sanjappa & Predeep [Fabaceae; Puerariae lobatae Radix]

*Pueraria montana* var. *lobata* (Willd.) Maesen and S.M.Almeida ex Sanjappa & Predeep [Fabaceae; *Puerariae lobatae* radix] is a common component of health products and medicines. Puerarin is a major iso-flavonoid extracted from this leguminous plant. *In vivo* study investigating the effect of puerarin on MCAO/R-induced rats shows that puerarin improves neurological deficit and reduces infarct size and brain water content in CI/RI rats. The probable mechanism proposed ias the involvement of puerarin in the TLR4/NF-κB signaling pathway (Zhou F. et al., 2014). *In vitro* study reports that puerarin and catalpol protect the brain from ischemia via protecting endothelial cells from apoptosis. This protection is related to HIF-1α that is dependent on the ERK and PI3K/AKT/mTOR signaling pathways (Liu Y. et al., 2017).

## 5 CHM Formulations for CI

### 5.1 Buyang Huanwu Tang

Buyang Huanwu Tang (BHT) is a classic formula that has been used in patients with ischemic stroke for many years. It originates from the old record “Yi Lin Gai Cuo” and was compiled by Qingren Wang, a famous doctor in the Qing dynasty. BHT contains six botanical drug(s), including *Astragalus mongholicus* Bunge [Fabaceae; *Astragali* radix]*, Angelica sinensis* (Oliv.) Diels [Apiaceae; *Angelicae sinensis* radix]*, Paeonia lactiflora* Pall [Paeoniaceae; *Paeoniae* radix rubra]*, Conioselinum anthriscoides* ‘Chuanxiong’ [Apiaceae; *Chuanxiong* rhizoma]*, Prunus persica* (L.) Batsch [Rosaceae; *Persicae* semen] and *Carthamus tinctorius* L [Asteraceae; *Carthami* Flos]*.* Reportedly, BHT can facilitate neurorehabilitation following a CI insult through improving synaptic plasticity (Pan et al., 2017). Chen *et al.* randomly divided 108 SD rats into sham operation, MCAO/R, MCAO/R + BHT, and MCAO/R + edaravone groups. They found that BHT treatment decreased cerebral edema and rat neurological function scores and reduced brain infarct volume by reducing the protein and mRNA levels of HIF-1α and VEGF (Chen Z. Z et al., 2019). In the study by Chen K. Y. et al. (2020), the authors reveal that BHT powdered product exhibited therapeutic efficacy for experimental stroke via reducing the expressions of TNF-α and IL-6 and increasing the expressions of TGF-β and IL-10. To explore the BHT effects on angiogenesis and neuroprotection after CI/RI, some researchers made H_2_O_2_/R-induced oxidative stress in human umbilical vein endothelial cells (HUVECs) and MCAO/R-induced rats. The data show that BHT promotes angiogenesis after CI/RI by controlling the Nox4/ROS and SIRT1/VEGF signaling pathways (Zheng et al., 2018; Shen et al., 2020).

### 5.2 Hulian Jiedu Tang

Hulian Jiedu Tang (HJT), an ancient antipyretic and detoxifying formula, is first recorded in the book “Wai Tai Mi Yao” by Wang Tao during the Tang Dynasty. It consists of *Coptis chinensis* Franch [Ranunculaceae; *Coptidis* rhizoma], *Scutellaria baicalensis* Georgi [Lamiaceae; *Scutellariae* radix], *Phellodendron amurense* Rupr [Rutaceae; *Phellodendri chinensis* cortex], and *Gardenia jasminoides* J. Ellis [Rubiaceae; *Gardeniae* fructus] in a ratio of 3:2:2:3. HJT is reported to improve antioxidant, anti-inflammatory, and antiapoptosis and regulate energy metabolism and mitochondrial function activities in CI/RI models. Zhu *et al.* applied metabolomics analysis and basic pathophysiology to evaluate the mechanisms of HJT against CI comprehensively. The data show that HJT can modulate metabolic stress, regulate the glutamate/GABA-glutamine cycle, and maintain cholinergic neuron function to modulate cerebral blood flow, regulate neuroinflammation and enhance memory (Zhu et al., 2018). Furthermore, HJT relieved CI/RI and neuronal apoptosis in MCAO/R-induced SD rats and OGD/R-induced cerebral cortical neurons through activating the PI3K/AKT signaling pathway and HIF-1α (Zhang Q. et al., 2014). In CI/RI rats, HJT induced protective autophagy by elevating the levels of Beclin-1 and LC3-II/LC3-I, inhibiting the mTOR via MAPK signaling pathway; in the other words, via restraining the phosphorylation of JNK and P38 and promoting the activation of ERK (Wang et al., 2013). A reporter explored the effects of the total alkaloids, flavonoids, and iridoids from HJT against CI/RI, and the result suggests that the total iridoids of HJT promote angiogenesis by modulating VEGF, Ang-1/-2, and TGF-β1; alkaloids were partially correlated with increased phosphorylation of AKT and GSK-3β; and flavonoids might be associated with the regulation of AKT, GSK-3β, mRNA, and Ang-1 protein levels (Zou et al., 2016). Berberine, baicalin, and geniposide (BBG), the three major ingredients in HJT, exhibit outstanding antioxidative effects for MCAO/R-injury rats by modulating the activities of SOD, CAT, and GPX (Fu et al., 2019).

### 5.3 Taohong Siwu Tang

Taohong Siwu Tang (TST), consists of *Prunus persica* (L.) Batsch [Rosaceae; *Persicae* semen], *Carthamus tinctorius* L [Asteraceae; *Carthami* Flos], *Angelica sinensis* (Oliv.) Diels [Apiaceae; *Angelicae sinensis* radix], *Conioselinum anthriscoides* ‘Chuanxiong’ [Apiaceae; *Chuanxiong* rhizoma], *Rehmannia glutinosa* (Gaertn.) DC [Orobanchaceae; *Rehmanniae* radix], and *Paeonia lactiflora* Pall [Paeoniaceae; *Paeoniae* radix rubra] and has been used clinically for the treatment of cerebrovascular diseases due to its effect of promoting blood circulation. TST reduces infarct volume in CI/RI rats through inhibiting inflammatory responses, apoptosis formation, and platelet activation via regulating the expressions of HIF-1α, TNF-α, iNOS, and Caspase-3 (Wu C. J. et al., 2011). TST modulates the expressions of brain-derived neurotrophic factor and p53 to play a role in treating and protecting CI/RI (Fan et al., 2015; Wu, 2018).

### 5.4 Nao Tai Fang

Nao Tai Fang (NTF) is a CHM formulation that improves blood circulation*.* The substance is formulated by modifying BHT and consists of *Astragalus mongholicus* Bunge [Fabaceae; *Astragali* Radix], *Conioselinum anthriscoides* ‘Chuanxiong’ [Apiaceae; *Chuanxiong* rhizoma], *Pheretima aspergillum* (E. Perrier) [Megascolecidae; Pheretima], and *Bombyx mori* Linnaeus [Bombycidae; Bombyx batryticatus]*.* Some studies demonstrate that NTF is clinically effective for the treatment of CI/RI, and the therapeutic mechanism is correlated with neuron ferroptosis*.* In an experimental investigation of NTF extraction on rat models of CI/RI, the author reports an increased expression of iron in the hippocampal CA2 region in the treatment group (Liao et al., 2015), suggesting the protective effect of NTF on the neurons by increasing the expression of iron and promoting neuronal iron efflux in CI/RI models. Furthermore, the recent study indicates that NTF extraction exerts a neuroprotective effect by promoting the expression of SLC7A11, intracellular GSH synthesis, and GPX4 activity and downregulating TFR1 and DMT1 protein levels in the MCAO/R-induced rats (Lan et al., 2020).

### 5.5 Qishen Yiqi

Qishen Yiqi (QY), composed of *Astragalus mongholicus* Bunge [Fabaceae; *Astragali* Radix], *Salvia miltiorrhiza* Bunge [Lamiaceae; *Salviae miltiorrhizae* radix et rhizoma], *Panax notoginseng* (Burkill) F.H.Chen [Araliaceae; *Notoginseng* radix et rhizoma], and *Dalbergia odorifera* T.C.Chen [Fabaceae; *Dalbergiae odoriferae* lignum], exerts the function of Qi tonifying and blood activating. The authors use the combination method of the pharmacology network and experimental verification to excavate the potential of QY about the reduction of neuroinflammatory response in a stroke subject. The result shows that the core targets associated with neuroinflammatory response were as follows: IL-1β, IL-6, TNF-α, NF-κB p65, TGF-β1, TLR4, and Interferon-γ (IFNG-γ). IFNG-γ is an inflammatory mediator released from CD4^+^ and CD8^+^ T-lymphocytes as well as natural killer (NK) cells (Wang Y. et al., 2020).

### 5.6 Shengnao Kang Decoction

Shengnao Kang Decoction (SKD) includes 15 kinds of CHM as follows: *Conioselinum anthriscoides* “Chuanxiong” [Apiaceae; *Chuanxiong* rhizoma], *Salvia miltiorrhiza* Bunge [Lamiaceae; *Salviae miltiorrhizae* radix et rhizoma], *Gastrodia elata* Blume [Orchidaceae; *Gastrodiae* rhizome], *Panax notoginseng* (Burkill) F.H.Chen [Araliaceae; *Notoginseng* radix et rhizoma], *Astragalus mongholicus* Bunge [Fabaceae; *Astragali* Radix], *Pueraria montana* var. *lobata* (Willd.) Maesen and S.M.Almeida ex Sanjappa and Predeep [Fabaceae; *Puerariae lobatae* radix], *Paeonia lactiflora* Pall [Paeoniaceae; *Paeoniae* radix rubra], *Leonurus japonicus* houtt. [Lamiaceae; *Leonuri* herba], *Uncaria rhynchophylla* (Miq.) Miq. [Rubiaceae; *Uncariae* ramulus cum uncis], *Sophora flavescens* Aiton [Fabaceae; *Sophora flavescentis* radix], *Whitmania pigra* Whitman [Hirudinidae; Hirudo], *Eupolyphaga sinensis* Walker [eupolyphaga; Eupolyphaga steleophaga], *Bombyx mori* Linnaeus [Bombycidae; Bombyx batryticatus], *Pheretima aspergillum* (E. Perrier) [Megascolecidae; Pheretima], and *Scolopendra subspinipes mutilans* L. Koch [Scolopendridae; Scolopendra]. It has efficacies of activating blood circulation and dissipating blood stasis, dredging meridians and collaterals. Dang *et al.* carried out a study on the antithrombotic effect of reduced SKD (RSKD), which is composed of *Conioselinum anthriscoides* “Chuanxiong” [Apiaceae; *Chuanxiong* rhizoma], *Salvia miltiorrhiza* Bunge [Lamiaceae; *Salviae miltiorrhizae* radix et rhizoma], *Astragalus mongholicus* Bunge [Fabaceae; *Astragali* Radix], *Pueraria montana* var. *lobata* (Willd.) Maesen and S.M.Almeida ex Sanjappa and Predeep [Fabaceae; *Puerariae lobatae* Radix], *Paeonia lactiflora* Pall [Paeoniaceae; *Paeoniae* radix rubra], and *Panax notoginseng* (Burkill) F.H.Chen [Araliaceae; *Notoginseng* radix et rhizoma]. RSKD had activities of anticoagulation, the regulation of active substances in vascular endothelium, and maintaining the balance of thromboxane A2 (TXA2) and prostaglandins I2 (PGI2) in CI/RI rats (Dang et al., 2015). In a study, the treatment with SKD on MCAO/R rats could diminish the levels of MDA, iNOS, and TNOS and increase the SOD and GPX activities (Chen et al., 2014).

### 5.7 Gualou Guizhi Decoction

Gualou Guizhi decoction (GGD), written by Zhang Zhongjing in “JinKui YaoLue” during the Eastern Han Dynasty, consists of six kinds of CHM: *Trichosanthes kirilowii* Maxim. [Cucurbitaceae; *Trichosanthis* fructus], *Neolitsea cassia* (L.) Kosterm. [Lauraceae; *Cinnamomi* ramulus], *Paeonia lactiflora* Pall [Paeoniaceae; *Paeoniae* radix rubra], *Zingiber officinale* Roscoe [Zingiberaceae; *Zingiberis* rhizoma], *Ziziphus jujuba* Mill [Rhamnaceae; *Jujubae* fructus], and *Glycyrrhiza uralensis* Fisch. ex DC [Fabaceae; *Glycyrrhizae* radix et rhizoma]. It has long been used to treat CI in a clinical setting in China. Some studies suggest that GGD has a neuroprotective effect on CI/RI models, and this effect is likely to be associated with its function as an antioxidant (by reducing MDA level and increasing GSH and SOD activities) (Zhang S. et al., 2015), anti-inflammatory (by regulating the NF-κB signaling pathway) (Hu et al., 2015), and antiapoptosis (by regulating protein expression and translocation in the PARP-1/AIF signaling pathway) (Nan et al., 2020; Hu et al., 2018). After the oral administration of 10 ml/kg GGD to MCAO/R model rats for 7 days, Hu *et al.* find that GGD can be against intracellular Ca^2+^ overload. Due to the effectiveness, GGD was made into the Gualou Guizhi granule. The Gualou Guizhi granule, as a hospital preparation of the second people’s Hospital of Fujian province (Min drug system approval No. S20130001) (Yang et al., 2012), is widely used for treating muscular spasticity following CI/RI. Zhang *et al.* find that Gualou Guizhi Granule protects oxidative injury by activating the Nrf2/antioxidant response element (ARE) signaling pathway in MCAO/R-induced rats and H_2_O_2_/R-induced PC12 cells (Zhang Y. et al., 2018).

### 5.8 Muxiang You Fang

Muxiang You Fang (MYF), one of the classical Traditional Hui Medicine (THM) formulas, was recorded in a Chinese Hui medical classic “Hui Hui Yao Fang.” It is regared to be beneficial in the treatment of CI/RI with consists of *Aucklandia costus* Falc. [Asteraceae; *Aucklandiae* radix], *Piper nigrum* L [Piperaceae; *Piperis* fructus], *Euphorbia pekinensis* Rupr. [Euphorbiaceae; *Euphorbiae pekinensis radix*], *Phoca vitulina* (L.) [Otariidae; *Phocae vitulina* testes et penis], and *Asarum sieboldii* Miq [Aristolochiaceae; *Asari* radix et rhizoma]*.* The group of Zhao QP found that MYF has potential neuroprotective activities by modulating the ratio between Bcl-2 and Bax (Zhao et al., 2016); promoting the protein expressions of Cyt-c and Caspase-3/-9 (Zhao et al., 2016); increasing the content of SOD, LDH, CAT, MDA, and GSH-PX (Wang et al., 2017); and activating the AMPK/mTOR signaling pathway (Chen, 2016) in treatment of CI/RI rats. THM, a branch of CHM, is efficient in the treatment of encephalopathy, especially stroke.

### 5.9 Shuan Tong Ling

Shuan Tong Ling (STL), a formula used to treat brain diseases, is composed of *Pueraria montana* var. *lobata* (Willd.) Maesen and S.M.Almeida ex Sanjappa and Predeep [Fabaceae; *Puerariae lobatae* radix], *Salvia miltiorrhiza* Bunge [Lamiaceae; *Salviae miltiorrhizae* radix et rhizoma], *Curcuma longa* L. [Zingiberaceae; *Curcumae longae* rhizoma], *Crataegus monogyna* Jacq [Rosaceae; *Crataegi* fructus], *Salvia chinensis* Benth. [Lamiaceae; *Salviae* herba], *Sinapis alba* L [Brassicaceae; *Sinapis* semen], *Astragalus mongholicus* Bunge [Fabaceae; *Astragali* radix], *Panax japonicus* (T.Nees) C.A.Mey [Araliaceae; *Panacis japonici* rhizoma], *Atractylodes macrocephala* Koidz. [Asteraceae; *Atractylodis macrocephalae* rhizoma], *Paeonia lactiflora* Pall. [Paeoniaceae; *Paeoniae* radix rubra], *Petroselinum crispum* (Mill.) Fuss [Apiaceae; Bupleuri radix], *Matricaria chamomilla* L. [Asteraceae; *Matricariae fols*]*, Cyperus rotundus* L. [Cyperaceae; *Cyperi* rhizoma], and *Gastrodia elata* Blume [Orchidaceae; *Gastrodiae* rhizome]. MCAO/R-induced SD rats revealed that STL exerts anti-inflammatory and antiapoptosis effects through activation of the SIRT1 signaling pathway (Mei et al., 2017).

## 6 CHM Preparations for CI/RI

### 6.1 Angong Niuhuang Wan

Angong Niuhuang Wan (ANW), one of the representative prescriptions for treating CI, originated from the “Wenbing Tiaobian” written by the physician Jutong Wu in the Qing Dynasty. It consists of *Coptis chinensis* Franch. [Ranunculaceae; *Coptidis* rhizoma], *Scutellaria baicalensis* Georgi [Lamiaceae; *Scutellariae* radix], *Gardenia jasminoides* J. Ellis [Rubiaceae; *Gardeniae* fructus], *Curcuma aromatica* Salisb. [Zingiberaceae; *Curcumae* radix], *Bos taurus domesticus* Gmeli [Bovidae; Bovis calculus], *Bubalus bubalis* Linnaeu [Bovidae; *Bubali* cornu], *Moschus berezovskii* Flerov [Cervidae; Moschus], *Pteria martensii* (Dunker) [Pteriidae; Margarita], *cinnabaris* (mineral drug), *sulfur* (mineral drug), and *borneolum syntheticum* (Guo Y. et al., 2014). The meta-analysis shows that ANW treatment improves the total response rate and neurologic deficit score among acute CI and intracerebral hemorrhage patients (Liu et al., 2019). A recent study uses MCAO/R-induced SD rats to explore the protective effects against CI/RI, and finds that the underlying mechanisms of ANW can be attributed to preserving BBB integrity by inhibiting the activation of MMP-2/-9 and upregulating the expressions of ZO-1 and claudin-5 (Tsoi et al., 2019a; Tsoi et al., 2019b). Even though there is a study reporting that *sulfur* and *cinnabaris* are essential components contributing to the neuroprotection of ANW with no hepatorenal toxicity in CI/RI, the arsenic- and mercury-containing ingredients in the formula still catch attention for causing health problems after consumption (Tsoi et al., 2019b). For safety and convenience, Zhang *et al.* extracted and purified components from ANW, removed *cinnabaris* and *sulfur*, and then made it to be an Angong Niuhuang (AN) sticker, which has the functions of activating blood circulation to dissipate blood stasis, cooling blood, and promoting the circulation of Qi to relieve pain. The more exciting thing is that the AN sticker combined with point application can improve cognitive function, promote the expression of Bcl-2, and inhibit the expressions of Bax and p53 in the hippocampal CA1 area of MCAO/R-induced rats and present a protective effect on neuronal damage (Zhang D. et al., 2012; Zhang R. et al., 2015).

### 6.2 Qingkai Ling Injection

Qingkai Ling Injection (QLI) is a patented CHM that is approved by the China FDA to treat ischemic stroke (http://samr.cfda.gov.cn/WS01/CL0412/). It is derived from ANW, whose main ingredients include *Panax ginseng* C.A.Mey. [Araliaceae; *Ginseng* radix et rhizoma], *Cornus mas* L. [Cornaceae; *Corni* fructus], *Scutellaria baicalensis* Georgi [Lamiaceae; *Scutellariae* radix], *Lonicera japonica* Thunb [Caprifoliaceae; *Lonicerae japonicae* flos], and *Gardenia jasminoides* J. Ellis [Rubiaceae; *Gardeniae* fructus]. QLI is widely used for treating ischemic stroke with the effects of heat clearing and detoxifying, eliminating phlegm and freeing channels, tranquilizing and allaying agitation, recovering consciousness, and keeping the balance of Qi and blood (Wu et al., 2014). Ma *et al.* did an experiment on MCAO/R-induced SD rats to assess the protective effect of treatment. As a result, QLI significantly inhibited inflammation by decreasing the levels of pro-inflammatory cytokines (TNF-α, IL-6, and IL-1β) and increasing the levels of anti-inflammatory cytokines (IL-4 and IL-10) and inhibited apoptosis and oxidative stress by regulating the MAPK signaling pathway (Ma et al., 2019). Subsequently, the authors inspected the neuroprotective effect of refined QLI (made up of baicalin, geniposide, cholic acid, and hyodeoxycholic acid) against CI/RI and detected that the effect was related to its attenuation of brain damage and cell apoptosis and activation of glial cells by modulation of the PI3K/Akt, TNF, NOD-like receptor, NF-κB, and TLR signaling pathways (Ma C. et al., 2020). Zhang *et al.* combined a network pharmacology–based approach with the experiment to probe the biological mechanisms of QLI against CI on the BBB and found that QLI alleviated BBB dysfunction, regulated expression of TJ, and modulated HIF-1α/MMP-9 activation (Zhang S. et al., 2020). With good efficacy and pharmacological effect, QLI is considered the first choice for the clinical treatment of CI/RI. Nevertheless, there are ADR reports regarding QKL in clinical practice (Wang et al., 2010). The medical staff and researchers should pay special attention to this problem.

### 6.3 Tongxin Luo Capsules

Tongxin Luo Capsules (TLC) are extracted and concentrated from 12 kinds of CHM, such as *Panax ginseng* C.A.Mey. [Araliaceae; *Ginseng* radix et rhizoma], *Paeonia lactiflora* Pall. [Paeoniaceae; *Paeoniae* radix rubra], *Santalum album* L. [Santalaceae; *Santali albi* lignum], Dalbergia odorifera *T.C.Chen [Leguminosae;* Dalbergiae odoriferae lignum]*,* Ziziphus jujuba *Mill. [Rhamnaceae;* Ziziphi jujubae *semen*]*, Whitmania pigra* Whitman [Hirudinidae; Hirudo], *Eupolyphaga sinensis* Walker [eupolyphaga; Eupolyphaga steleophaga, *Buthus martensii* Karsch [Buthidae; Scorpio], *Scolopendra subspinipes mutilans* L. Koch [Scolopendridae; Scolopendra], *Cryptotympana pustulata* Fabriciu [Cicadidae; Cicadae periostracum], *Boswellia carterii* Birdw [Burseraceae; Olibanum], and Borneolum syntheticum. They were approved by the FDA of China in 1996 for the treatment of ischemic stroke. The current research suggests that TLC inhibits neuronal apoptosis by modulating the PI3K/Akt (Yu et al., 2019) and Connexin 43/Calpain II/Bax/Caspase-3 signaling pathways (Cheng et al., 2017); it inhibits postischemic inflammation by downregulating AQP4 expression and inhibiting the activation of high mobility group box 1 (one of the major inflammatory mediators), TLR4, NF-κB, and TNF-α (Cai et al., 2016). It also lowers cerebral infarct volume (Cheng et al., 2014), enhances neurogenesis as well as angiogenesis (Chen, 2016), and protects the brain from BBB disruption and increased TJ proteins (Li et al., 2015).

### 6.4 Dan Hong Injection

Dan Hong injection (DHI), a well-established CHM prescription, consists of *Salvia miltiorrhiza* Bunge [Lamiaceae; *Salviae miltiorrhizae* radix et rhizoma] and Carthamus *tinctorius* L. [Asteraceae; Carthamus *flos*]. It is applied extensively in clinical practice and shows significant therapeutic effects to CI/RI patients with the functions of promoting blood circulation and resolving stasis. Some researchers confirm the neuroprotective effects of DHI to CI/RI models and further discovered that these effects on the brain are partly generated by activating the PI3K-Akt (Feng et al., 2020), NF-κB (Jiang and Lian, 2015), and Nrf2/ARE signaling pathways (Guo H. et al., 2014) and enhancing the mitochondrial function (Orgah et al., 2019). As a CHM injection, DHI has a very good safety record, whose incidence rate of ADR was 3.50‰ (Orgah et al., 2019).

### 6.5 Pien Tze Huang

Pien Tze Huang (PTH), a famous and precious Chinese patent medicine in China and Southeast Asian countries, was formulated by a royal physician in the Ming Dynasty. It is made up of *Panax notoginseng* (Burkill) F.H.Chen [Araliaceae; *Notoginseng* radix et rhizoma], *Bos taurus domesticus* Gmeli [Bovidae; Bovis calculus] (animal drug), *Moschus berezovskii* Flerov [Cervidae; Moschus] (animal drug), *Panax ginseng* C.A.Mey. [Araliaceae; *Ginseng* radix et rhizoma], and *snake gall*. PTH has activities of heat clearing and detoxifying, promoting blood circulation, reducing blood stasis and swelling, and relieving pain. It shows preventive effects on limiting the damage or injury caused by CI/RI in rats. PTH inhibits mitochondria-mediated neuronal apoptosis and attenuates inflammatory responses by downregulating cytosolic Cyt-c, Bax, P53, and Caspase-3/-9 levels and upregulating levels of mitochondrial Cyt-c, Bcl-xl, and phosphorylation of AKT and GSK-3β (Zhang X. et al., 2018).

### 6.6 Zhong Feng Gao

Zhong Feng Gao (ZFG), a hospital preparation with the effects of replenishing Qi, promoting blood circulation, dispersing blood stasis, and dredging collateral, consists of *Curcuma longa* L. [Zingiberaceae; *Curcumae longae* rhizoma], *Conioselinum anthriscoides* “Chuanxiong” [Apiaceae; *Chuanxiong* rhizoma], *Astragalus mongholicus* Bunge [Fabaceae; *Astragali* radix], *Salvia miltiorrhiza* Bunge [Lamiaceae; *Salviae miltiorrhizae* radix et rhizoma], *Paeonia lactiflora* Pall. [Paeoniaceae; *Paeoniae* radix rubra], and *Glycyrrhiza uralensis* Fisch. ex DC [Fabaceae; *Glycyrrhizae* radix et rhizoma]. Huang et al. (2019) prepared ZFG-medicated serum through administrating ZFG (1 ml/100 g) and adding DMEM containing 10% ZFG-medicated serum to the reoxygenation medium. The result demonstrates that treatment with ZFG-medicated serum markedly alleviated OGD/R-induced BMEC injury by protecting angiogenesis via the Notch and Wnt signaling pathways.

### 6.7 Houshi Hei San

Houshi Hei San (HHS), produced by Zhongjing Zhang for the treatment of CI, is proved valuable in clinical practice, and it is composed of *Chrysanthemum×morifolium* (Ramat.) Hemsl. [Asteraceae; *Chrysanthemi* flos], *Saposhnikovia divaricata* (Turcz. ex Ledeb.) Schischk. [Apiaceae; *Saposhnikoviae* radix], *Neolitsea cassia* (L.). Kosterm [Lauraceae; *Cinnamomi* ramulus], *Conioselinum anthriscoides* “Chuanxiong” [Apiaceae; *Chuanxiong* rhizoma], *Asarum sieboldii* Miq.[ Aristolochiaceae; *Asari* radix et rhizoma], *Platycodon grandiflorus* (Jacq.) A. DC. [Campanulaceae; *Platycodonis* radix], *Atractylodes macrocephala* Koidz. [Asteraceae; *Atractylodis macrocephalae* rhizoma], *Zingiber officinale* Roscoe [Zingiberaceae; *Zingiberis* rhizoma]*, Angelica sinensis* (Oliv.) Diels [Apiaceae; *Angelicae sinensis* radix]*, Panax ginseng* C.A.Mey. (Araliaceae; *Ginseng* radix et rhizoma), *Scutellaria baicalensis* Georgi [Lamiaceae; *Scutellariae* radix], *Poria cocos* (Schw) Wolf [Polyporaceae; Poria], and *Ostrea gigas* Thunberg [Ostreidae; *Ostreae* concha]*.* HHS suppresses CI/RI by promoting astrocyte activation and diminishing inflammatory factor expression (Zhang Q. et al., 2012). It promotes angiogenesis via upregulating the expression of HIF-1α, VEGFA, and Ang-1 and downregulating the expression of Ang-2 (Xiang et al., 2019). HHS also can improve neurological function and survival by the activation of brain-derived neurotrophic factor (BDNF)/PI3K/Akt signaling pathways (Chang et al., 2016).

### 6.8 Sheng Mai Injection

Sheng Mai Injection (SMI), a derivative form of Sheng Mai San, consists of *Panax ginseng* C.A.Mey.(Araliaceae; *Ginseng* radix et rhizoma), *Ophiopogon japonicus* (Thunb.) Ker Gawl. [Asparagaceae; *Ophiopogonis* radix], and *Schisandra chinensis* (Turcz.) Baill. [Schisandraceae; *Schisandrae chinensis* fructus] and shows significant efficacy for the prevention and treatment of cerebrovascular diseases clinically. An *in vivo* study investigated the effect of SMI in MCAO/R rats at three oral doses: 1.42, 2.84, and 5.68 g/kg, which revealed that SMI attenuates CI/RI-induced autophagy by modulation of the AMPK, mTOR, and JNK signaling pathways (Yang et al., 2016). SMI maintained BBB integrity following CI/RI via regulating the expression and trafficking of TJ-associated proteins in lipid rafts (Xu, 2019).

### 6.9 Yiqi Fumai Powder Injection

Yiqi Fumai Powder Injection (YFPI), widely used for the treatment of cerebrovascular diseases, is made up of *Panax ginseng* C.A.Mey.(Araliaceae; *Ginseng* radix et rhizoma), *Ophiopogon japonicus* (Thunb.) Ker Gawl. [Asparagaceae; *Ophiopogonis* radix], and *Schisandra chinensis* (Turcz.) Baill. [Schisandraceae; *Schisandrae chinensis* fructus]. Some studies report that YFPI provides a neuroprotective effect against CI/RI in mice by improving BBB dysfunction via increasing the expression of TJ proteins (Cao et al., 2016) and inhibiting the activation of the NF-κB signaling pathway (Pan et al., 2020).

### 6.10 Xue Sai Tong Injection

Xue Sai Tong Injection (XSTI) is also a classic CHM injection; its main constituent is saponins, which are extracted from *Panax notoginseng* (Burkill) F.H.Chen [Araliaceae; *Notoginseng* radix et rhizoma]. The research finds that XSTI reduces neurological dysfunction and pathological damage, promotes weight gain and synaptic regeneration, and reduces Nogo-A mRNA and protein levels by inhibiting the ROCKII pathway in models of MCAO-induced rats and OGD/R-induced SH-SY5Y cells (Zhou et al., 2021).

## 7 Discussion

### 7.1 Summary of Results

In summary, CI/RI is a complex pathophysiological process. Different processes lead to different severity levels of CI/RI, among which the numerous factors and mechanisms interrelate with each other and influence mutually, resulting in cell apoptosis or nerve necrosis finally. Although remarkable progress has been made in the prevention and treatment of CI/RI in the past few decades, CI/RI remains a serious global health problem, implying that it remains an unmet clinical need for developing innovative drugs for CI/RI.

CHM has a polypharmacological effect naturally based on the “multi-components, multi-targets and multi-pathways” principle (Guo et al., 2020; Wang et al., 2021). Based on target molecules, biological function, and bioactive compounds, current developments in network pharmacology establish an intricate interaction network that matches the natural characteristics of CHM and provides the detailed action mechanism of CHM against CI/RI at the molecular level with a systematic viewpoint. We review senkyunolide-H from *Conioselinum anthriscoides* “Chuanxiong” [Apiaceae; *Chuanxiong* rhizoma], *Carthamus tinctorius* L. (CTL) [Asteraceae; *Carthami* Flos] extract/phospholipid complex, and *Styrax benzoin* Dryand. [Styracaceae; Benzoinum] in the paper (Zhou F. et al., 2014; Zhang Y. et al., 2018; Chen H. et al., 2020a). Furthermore, the application of various omics technologies, such as genomics and metabolomics, help investigators discover CI/RI drugs from CHM more comprehensively and systematically. The omics technologies are proved to be an efficient and robust way of identifying mechanisms of drug action and potential biomarkers and also afford prolific information to better understand the molecular pathogenesis of CI/RI (Liao et al., 2016; Liu S. Y. et al., 2017; Li et al., 2018; Wang R. et al., 2019). By integrating network pharmacology with metabolomics, researchers elucidate the action of CHM through constructing the “disease-targets-drug” network and screening out the nodes of particular interest, then focusing on the alteration of endogenous metabolites in biosamples to discover the underlying mechanism of CI/RI. The strategy combines network pharmacology with omics or some advanced analysis methods and can lay a considerable foundation for explicating the effect mechanism of CHM against CI/RI and then promoting its clinical application.

### 7.2 Limitations

Although many studies are missed in this review due to our search strategy and the limited access to some articles, the abovementioned evidence is strongly indicative that CHM is the emerging medicine in the prevention and/or treatment of CI/RI. However, some objective limitations should be considered based on the existing literature. First, many of the clinical trials are not well designed and have poor methodological quality by lacking formal inclusion/exclusion criteria, or the randomization procedures were inadequately described, the duration of therapy was too short, and conclusive results were unsatisfactory. Poorly designed and reported clinical trials usually exaggerate the treatment effects, which mislead decision making clinically. Second, CHM remedies used today have not undergone careful scientific assessment, and their preclinical studies as side effects, toxic effects, and major drug-to-drug interactions still lack in the record. Third, most of the components in CHM cannot go into the brain because they are not permeable through the BBB. The final concentration of those CHM components is very low compared with the *in vitro* concentration that shows some effects on cell models. Fourth, ingredients contained in one CHM, even in the extraction of a single CHM, are very complicated. Thus, the characteristics of multicomponent, multitarget, and slow onset in CHM are not conducive to the interpretation of the action mechanism for treating CI/RI and the interaction with other medicines (including western medicines). Additionally, most previous studies on the brain protective effects of CHM are conducted in young and healthy animals, which may not simulate the prevalence of stroke in elderly patients.

### 7.3 Outlooks

Regardless, CHM deserves more attention and application. Further study should combine the holistic concept of TCM theory with modern medicine and integrate different CHM to achieve the optimum benefit to counter nerve injury following CI/IR challenge. For example, a recent report suggests that the combined treatment of Ginkgo biloba dispersible tablets with nimodipine are widely used in treating CI/RI (Han et al., 2020). Furthermore, make full use of the integrated technologies, such as network pharmacology with omics or other advanced technologies to take some experimental research, searching the key part of action in CI/RI and the specific targets of CHM at a single compound level and discovering more cerebrovascular drugs from CHM. Omics technologies can characterize the molecular changes that underlie the development and progression of various complex human diseases and provide researchers with a greater understanding of the flow of information from the original cause of the disease (genetic, environmental, or developmental) to the functional consequences or relevant interactions. These technologies are proposed and heralded as the key to advancing precision medicine in the clinic (Hasin et al., 2017; Olivier et al., 2019). In the field of CI/RI, omics help reveal several key mechanisms in CI/RI development and discover compounds from CHM with the bioactivities for minimizing ischemic brain injury. These compounds include sesamin, baicalin, salvianolic acid A, 6-paradol, silymarin, apocynin, 3H-1,2-Dithiole-3-thione (-)-epicatechin, rutin, Dl-3-N-butylphthalide, and naringin (Chen H. et al., 2020b). Last but not least, the mechanisms of CI/RI in humans and experimental animals cannot be equal because of species differences. This paper presents an overview on the neuroprotective effect of CHM in experimental models. Therefore, whether CHM can exert the same protective mechanism in clinical practice requires further study. The clinical study criteria should be documented to standardize the evaluation of CHM. Currently, except for Xingnaojing injection, Naoxuekang, Xinnaoshutong, and Xuesaitong capsules (Chen et al., 2017; Lai et al., 2017), most CHM remedies used in the clinic have no ethics and dissemination registration number and trial registration number, and the trials did not follow the principles of randomization and blindness; besides this, the number of clinical samples was far from enough. These CHMs need reasonable and scientific clinical trials, and we call for more high-quality studies to confirm the neuroprotective effect and mechanism of CHM in others animal models such as dogs or rabbits in the future. In addition, international collaboration may be encouraged, promoted, and financed by the governments to improve the overall research quality. In this case, it is a promise to develop new drugs with effectiveness and safety from CHM for the prevention and treatment of patients with CI/RI.

## 8 Conclusion

Taken together, it cannot be ignored that CHM exerts a significant neuroprotective effect even when used alone in *in vivo* and *in vitro* studies. The results of various animal experiments further indicate that CHM has an intervention effect in inflammation, oxidative stress, apoptosis, Ca^2+^ overload, autophagy, and many others as well as the major signaling pathways in CI/RI, including PI3K/AKT, MAPKs, NF-κB, Nrf2, and others. Significant progress has been made in the studies of CHM as promising drug candidates for the treatment of CI/IR. In particular, they have been gaining significant interest recently in the stroke research field given the fact that no effective neuroprotective medicine is currently available. Future studies should pay more attention to assess CHM that offers the best and long-term CI/RI protection as well as functional and survival rate improvement using large and different rodent animal stroke models.

## References

[B1] An P. Xie J. Qiu S. Liu Y. Wang J. Xiu X. (2019). Hispidulin Exhibits Neuroprotective Activities against Cerebral Ischemia Reperfusion Injury through Suppressing NLRP3-Mediated Pyroptosis. Life Sci. 232, 116599. 10.1016/j.lfs.2019.116599 31247210

[B2] Anisimov A. Tvorogov D. Alitalo A. Leppänen V. M. An Y. Han E. C. (2013). Vascular Endothelial Growth Factor-Angiopoietin Chimera with Improved Properties for Therapeutic Angiogenesis. Circulation 127, 424–434. 10.1161/CIRCULATIONAHA.112.127472 23357661

[B3] Anrather J. Iadecola C. (2016). Inflammation and Stroke: An Overview. Neurotherapeutics 13, 661–670. 10.1007/s13311-016-0483-x 27730544 PMC5081118

[B4] Auriel E. Bornstein N. M. (2010). Neuroprotection in Acute Ischemic Stroke-Ccurrent Status. J. Cel Mol Med 14, 2200–2202. 10.1111/j.1582-4934.2010.01135.x PMC3822558 20716132

[B5] Bai X. Tan T. Y. Li Y. X. Li Y. Chen Y. F. Ma R. (2020). The Protective Effect of Cordyceps Sinensis Extract on Cerebral Ischemic Injury via Modulating the Mitochondrial Respiratory Chain and Inhibiting the Mitochondrial Apoptotic Pathway. Biomed. Pharmacother. 124, 109834. 10.1016/j.biopha.2020.109834 31978767

[B6] Barrington J. Lemarchand E. Allan S. M. (2017). A Brain in Flame; Do Inflammasomes and Pyroptosis Influence Stroke Pathology? Brain Pathol. 27, 205–212. 10.1111/bpa.12476 27997059 PMC8028888

[B7] Berliocchi L. Bano D. Nicotera P. (2005). Ca2+ Signals and Death Programmes in Neurons. Philos. Trans. R. Soc. Lond. B Biol. Sci. 360, 2255–2258. 10.1098/rstb.2005.1765 16321795 PMC1569591

[B8] Boehncke W. H. (2018). Systemic Inflammation and Cardiovascular Comorbidity in Psoriasis Patients: Causes and Consequences. Front. Immunol. 9, 579. 10.3389/fimmu.2018.00579 29675020 PMC5895645

[B9] Cai M. Guo Y. Wang S. Wei H. Sun S. Zhao G. (2017). Tanshinone IIA Elicits Neuroprotective Effect through Activating the Nuclear Factor Erythroid 2-Related Factor-dependent Antioxidant Response. Rejuvenation Res. 20, 286–297. 10.1089/rej.2016.1912 28162056

[B10] Cai M. Yu Z. Wang L. Song X. Zhang J. Zhang Z. (2016). Tongxinluo Reduces Brain Edema and Inhibits post-ischemic Inflammation after Middle Cerebral Artery Occlusion in Rats. J. Ethnopharmacol 181 (2), 136–145. 10.1016/j.jep.2016.01.026 26805468

[B11] Cao G. Ye X. Xu Y. Yin M. Chen H. Kou J. (2016). YiQiFuMai Powder Injection Ameliorates Blood-Brain Barrier Dysfunction and Brain Edema after Focal Cerebral Ischemia-Reperfusion Injury in Mice. Drug Des. Devel Ther. 10, 315–325. 10.2147/DDDT.S96818 PMC4716731 26834461

[B12] Catanese L. Tarsia J. Fisher M. (2017). Acute Ischemic Stroke Therapy Overview. Circ. Res. 120, 541–558. 10.1161/CIRCRESAHA.116.309278 28154103

[B13] Chang J. Yao X. Zou H. Wang L. Lu Y. Zhang Q. (2016). BDNF/PI3K/Akt and Nogo-A/RhoA/ROCK Signaling Pathways Contribute to Neurorestorative Effect of Houshiheisan against Cerebral Ischemia Injury in Rats. J. Ethnopharmacol 194 (24), 1032–1042. 10.1016/j.jep.2016.11.005 27833029

[B14] Chang L. Yin C. Y. Wu H. Y. Tian B. B. Zhu Y. Luo C. X. (2017). (+)-Borneol Is Neuroprotective against Permanent Cerebral Ischemia in Rats by Suppressing Production of Proinflammatory Cytokines. J. Biomed. Res. 31, 306–314. 10.7555/JBR.31.20160138 28808202 PMC5548991

[B15] Chang L. L. Li C. Li Z. L. Wei Z. L. Jia X. B. Pang S. T. (2020). Carthamus tinctorius L. Extract Ameliorates Cerebral Ischemia-Reperfusion Injury in Rats by Regulating Matrix Metalloproteinases and Apoptosis. Indian J. Pharmacol. 52 (2), 108–116. Epub 2020 Jun 3. PMID: 32565598; PMCID: PMC7282686. 10.4103/ijp.IJP_400_18 32565598 PMC7282686

[B16] Chen A. L. (2016). Protective Effect of Mu-Xiang-You-Fang on PC12 Cell Injury Induced by Oxygen-Glucose Deprivation and Reperfusion Based on AMPK/mTOR Signaling Pathway. Ningxia, China: Master’s Degree of Ningxia Medical University.

[B204] Chen H. Cao H. Guo X. Zhao M. Xia Q. Chen B. (2017). Naoxuekang, Xinnaoshutong and Xuesaitong capsules for treating stroke: a protocol for a randomised controlled trial. BMJ Open 7(11), e015983. 10.1136/bmjopen-2017-015983 PMC5695516 29122785

[B17] Chen H. He Y. Chen S. Qi S. Shen J. (2020b). Therapeutic Targets of Oxidative/nitrosative Stress and Neuroinflammation in Ischemic Stroke: Applications for Natural Product Efficacy with Omics and Systemic Biology. Pharmacol. Res. 158, 104877. 10.1016/j.phrs.2020.104877 32407958

[B18] Chen H. Ren M. Li H. Xie Q. Ma R. Li Y. (2020a). Neuroprotection of Benzoinum in Cerebral Ischemia Model Rats via the ACE-AngI-VEGF Pathway. Life Sci. 260, 118418. 10.1016/j.lfs.2020.118418 32931799

[B19] Chen K. Y. Wu K. C. Hueng D. Y. Huang K. F. Pang C. Y. (2020). Anti-inflammatory Effects of Powdered Product of Bu Yang Huan Wu Decoction: Possible Role in Protecting against Transient Focal Cerebral Ischemia. Int. J. Med. Sci. 17 (12), 1854–1863. 10.7150/ijms.46581 32714088 PMC7378667

[B20] Chen L. Wang X. Zhang J. Dang C. Liu G. Liang Z. (2016). Tongxinluo Enhances Neurogenesis and Angiogenesis in Peri-Infarct Area and Subventricular Zone and Promotes Functional Recovery after Focal Cerebral Ischemic Infarction in Hypertensive Rats. Evid. Based Complement. Alternat Med. 2016, 8549590. 10.1155/2016/8549590 27069496 PMC4812278

[B21] Chen L. Xiang Y. Kong L. Zhang X. Sun B. Wei X. (2013). Hydroxysafflor Yellow A Protects against Cerebral Ischemia-Reperfusion Injury by Anti-apoptotic Effect through PI3K/Akt/GSK3β Pathway in Rat. Neurochem. Res. 38 (11), 2268–2275. 10.1007/s11064-013-1135-8 23990223

[B22] Chen L. Zhao Y. Zhang T. Dang X. Xie R. Li Z. (2014). Protective Effect of Sheng-Nao-Kang Decoction on Focal Cerebral Ischemia-Reperfusion Injury in Rats. J. Ethnopharmacol 151, 228–236. 10.1016/j.jep.2013.10.015 24161430

[B23] Chen S. Peng H. Rowat A. Gao F. Zhang Z. Wang P. (2015). The Effect of Concentration and Duration of Normobaric Oxygen in Reducing Caspase-3 and -9 Expression in a Rat-Model of Focal Cerebral Ischaemia. Brain Res. 1618, 205–211. 10.1016/j.brainres.2015.05.027 26032740

[B24] Chen X X. Wu H. Chen H. Wang Q. Xie X. J. Shen J. (2019). Astragaloside VI Promotes Neural Stem Cell Proliferation and Enhances Neurological Function Recovery in Transient Cerebral Ischemic Injury via Activating EGFR/MAPK Signaling Cascades. Mol. Neurobiol. 56, 3053–3067. 10.1007/s12035-018-1294-3 30088176

[B25] Chen Z. Z Z.-Z. Gong X. Guo Q. Zhao H. Wang L. (2019). Bu Yang Huan Wu Decoction Prevents Reperfusion Injury Following Ischemic Stroke in Rats via Inhibition of HIF-1 α, VEGF and Promotion β-ENaC Expression. J. Ethnopharmacology 228, 70–81. 10.1016/j.jep.2018.09.017 30218809

[B26] Cheng Q. Tong F. Shen Y. He C. Wang C. Ding F. (2019). Achyranthes Bidentata Polypeptide K Improves Long-Term Neurological Outcomes through Reducing Downstream Microvascular Thrombosis in Experimental Ischemic Stroke. Brain Res. 1706, 166–176. 10.1016/j.brainres.2018.11.010 30414726

[B27] Cheng X. Hou Z. Sun J. Huang Y. Wang L. Zhou Z. (2017). Protective Effects of Tongxinluo on Cerebral Ischemia/reperfusion Injury Related to Connexin 43/Calpain II/Bax/Caspase-3 Pathway in Rat. J. Ethnopharmacol 198, 148–157. 10.1016/j.jep.2017.01.004 28065778

[B28] Cheng X. Luo H. Zhou L. Wang L. Sun J. Huang Y. (2014). Neuroprotective Effect of the Traditional Chinese Herbal Formula Tongxinluo: a PET Imaging Study in Rats. Neural Regen. Res. 9 (13), 1267–1274. 10.4103/1673-5374.137573 25221578 PMC4160852

[B29] Dang X. Miao J. J. Chen A. Q. Li P. Chen L. Liang J. R. (2015). The Antithrombotic Effect of RSNK in Blood-Stasis Model Rats. J. Ethnopharmacol 173 (173), 266–272. 10.1016/j.jep.2015.06.030 26216512

[B30] Dawson T. M. Dawson V. L. (2017). Mitochondrial Mechanisms of Neuronal Cell Death: Potential Therapeutics. Annu. Rev. Pharmacol. Toxicol. 57, 437–454. 10.1146/annurev-pharmtox-010716-105001 28061689 PMC11323062

[B31] Dong X. Gao J. Zhang C. Y. Hayworth C. Frank M. Wang Z. (2019). Neutrophil Membrane-Derived Nanovesicles Alleviate Inflammation to Protect Mouse Brain Injury from Ischemic Stroke. ACS Nano 13, 1272–1283. 10.1021/acsnano.8b06572 30673266 PMC6424134

[B32] Du C. P. Tan R. Hou X. Y. (2012). Fyn Kinases Play a Critical Role in Neuronal Apoptosis Induced by Oxygen and Glucose Deprivation or Amyloid-β Peptide Treatment. CNS Neurosci. Ther. 18, 754–761. 10.1111/j.1755-5949.2012.00357.x 22709448 PMC6493628

[B33] Fan R. J. Luo Y. F. Chen Y. S. He G. Z. Tang Z. S. Lu Y. (2015). The Effects of Taohong Siwu Tang on the Caspase-3 and P53 Expression in Cerebral Cortex Neurons of Rat Cerebral Ischemia Reperfusion Injury Model. Chin. J. Neur 31 (06), 739–745. 10.16557/j.cnki.1000-7547.2015.06.0012

[B34] Fang X. Li Y. Qiao J. Guo Y. Miao M. (2017). Neuroprotective Effect of Total Flavonoids from Ilex Pubescens against Focal Cerebral Ischemia/reperfusion Injury in Rats. Mol. Med. Rep. 16, 7439–7449. 10.3892/mmr.2017.7540 28944915 PMC5865877

[B35] Feng C. Wan H. Zhang Y. Yu L. Shao C. He Y. (2020). Neuroprotective Effect of Danhong Injection on Cerebral Ischemia-Reperfusion Injury in Rats by Activation of the PI3K-Akt Pathway. Front. Pharmacol. 11, 298. 10.3389/fphar.2020.00298 32218735 PMC7078680

[B36] Fu P. K. Pan T. L. Yang C. Y. Jeng K. C. Tang N. Y. Hsieh C. L. (2016). Carthamus tinctorius L. Ameliorates Brain Injury Followed by Cerebral Ischemia-Reperfusion in Rats by Antioxidative and Anti-inflammatory Mechanisms. Iran J. Basic Med. Sci. 19, 1368–1375. 10.22038/ijbms.2016.7925 28096971 PMC5220244

[B37] Fu X. Wang J. Liao S. Lv Y. Xu D. Yang M. (2019). 1H NMR-Based Metabolomics Reveals Refined-Huang-Lian-Jie-Du-Decoction (BBG) as a Potential Ischemic Stroke Treatment Drug with Efficacy and a Favorable Therapeutic Window. Front. Pharmacol. 10, 337. 10.3389/fphar.2019.00337 31031621 PMC6474285

[B38] Geng X. Parmar S. Li X. Peng C. Ji X. Chakraborty T. (2013). Reduced Apoptosis by Combining Normobaric Oxygenation with Ethanol in Transient Ischemic Stroke. Brain Res. 1531, 17–24. 10.1016/j.brainres.2013.07.051 23920008

[B39] Guan X. Li Z. Zhu S. Cheng M. Ju Y. Ren L. (2021). Galangin Attenuated Cerebral Ischemia-Reperfusion Injury by Inhibition of Ferroptosis through Activating the SLC7A11/GPX4 axis in Gerbils. Life Sci. 264, 118660. 10.1016/j.lfs.2020.118660 33127512

[B40] Guo F. Tang X. Zhang W. Wei J. Tang S. Wu H. (2020). Exploration of the Mechanism of Traditional Chinese Medicine by AI Approach Using Unsupervised Machine Learning for Cellular Functional Similarity of Compounds in Heterogeneous Networks, XiaoErFuPi Granules as an Example. Pharmacol. Res. 160, 105077. 10.1016/j.phrs.2020.105077 32687952

[B41] Guo H. Li M. J. Liu Q. Q. Guo L. L. Ma M. M. Wang S. X. (2014). Danhong Injection Attenuates Ischemia/reperfusion-Induced Brain Damage Which Is Associating with Nrf2 Levels *In Vivo* and *In Vitro* . Neurochem. Res. 39 (9), 1817–1824. 10.1007/s11064-014-1384-1 25069640

[B42] Guo R. B. Wang G. F. Zhao A. P. Gu J. Sun X. L. Hu G. (2012). Paeoniflorin Protects against Ischemia-Induced Brain Damages in Rats via Inhibiting MAPKs/NF-Κb-Mediated Inflammatory Responses. PLoS One 7, e49701. 10.1371/journal.pone.0049701 23166749 PMC3498223

[B43] Guo Y. Yan S. Xu L. Zhu G. Yu X. Tong X. (2014). Use of Angong Niuhuang in Treating central Nervous System Diseases and Related Research. Evid. Based Complement. Alternat Med. 2014, 346918. 10.1155/2014/346918 25587341 PMC4281447

[B44] Han J. Xiao Q. Lin Y. H. Zheng Z. Z. He Z. D. Hu J. (2015). Neuroprotective Effects of Salidroside on Focal Cerebral Ischemia/reperfusion Injury Involve the Nuclear Erythroid 2-related Factor 2 Pathway. Neural Regen. Res. 10, 1989–1996. 10.4103/1673-5374.172317 26889188 PMC4730824

[B45] Han J. Xu H. H. Chen X. L. Hu H. R. Hu K. M. Chen Z. W. (2018). Total Flavone of Rhododendron Improves Cerebral Ischemia Injury by Activating Vascular TRPV4 to Induce Endothelium-Derived Hyperpolarizing Factor-Mediated Responses. Evid. Based Complement. Alternat Med. 2018, 8919867. 10.1155/2018/8919867 30405745 PMC6201489

[B46] Han K. Rong W. Wang Q. Qu J. Li Q. Bi K. (2020). Time-dependent Metabolomics Study of Cerebral Ischemia-Reperfusion and its Treatment: Focus on the Combination of Traditional Chinese Medicine and Western Medicine. Anal. Bioanal. Chem. 412 (26), 7195–7209. 10.1007/s00216-020-02852-w 32783128

[B47] Hasin Y. Seldin M. Lusis A. (2017). Multi-omics Approaches to Disease. Genome Biol. 18 (1), 83. 10.1186/s13059-017-1215-1 28476144 PMC5418815

[B48] He Q. Sun J. Wang Q. Wang W. He B. (2014). Neuroprotective Effects of Ginsenoside Rg1 against Oxygen-Glucose Deprivation in Cultured Hippocampal Neurons. J. Chin. Med. Assoc. 77, 142–149. 10.1016/j.jcma.2014.01.001 24548377

[B49] Hou K. Xu D. Li F. Chen S. Li Y. (2019). The Progress of Neuronal Autophagy in Cerebral Ischemia Stroke: Mechanisms, Roles and Research Methods. J. Neurol. Sci. 400, 72–82. 10.1016/j.jns.2019.03.015 30904689

[B50] Hu G. Q. Du X. Li Y. J. Gao X. Q. Chen B. Q. Yu L. (2017). Inhibition of Cerebral Ischemia/reperfusion Injury-Induced Apoptosis: Nicotiflorin and JAK2/STAT3 Pathway. Neural Regen. Res. 12 (1), 96–102. 10.4103/1673-5374.198992 28250754 PMC5319249

[B51] Hu H. X. Lin R. H. Zhu X. Q. Li Z. F. Chen L. D. (2015). Anti-inflammatory Effects of Gualou Guizhi Decoction in Transient Focal Cerebral Ischemic Brains. [Corrected]. Mol. Med. Rep. 12 (1), 1321–1327. 10.3892/mmr.2015.3511 25815521

[B52] Hu J. Pang W. S. Han J. Zhang K. Zhang J. Z. Chen L. D. (2018). Gualou Guizhi Decoction Reverses Brain Damage with Cerebral Ischemic Stroke, Multi-Component Directed Multi-Target to Screen Calcium-Overload Inhibitors Using Combination of Molecular Docking and Protein-Protein Docking. J. Enzyme Inhib. Med. Chem. 33, 115–125. 10.1080/14756366.2017.1396457 29185359 PMC6009878

[B53] Huang S. Gong T. Zhang T. Wang X. Cheng Q. Li Y. (2019). Zhongfenggao Protects Brain Microvascular Endothelial Cells from Oxygen-Glucose Deprivation/Reoxygenation-Induced Injury by Angiogenesis. Biol. Pharm. Bull. 42, 222–230. 10.1248/bpb.b18-00650 30518742

[B54] Ji K. Xue L. Cheng J. Bai Y. (2016). Preconditioning of H2S Inhalation Protects against Cerebral Ischemia/reperfusion Injury by Induction of HSP70 through PI3K/Akt/Nrf2 Pathway. Brain Res. Bull. 121, 68–74. 10.1016/j.brainresbull.2015.12.007 26772627

[B55] Jia Y. Zuo D. Li Z. Liu H. Dai Z. Cai J. (2014). Astragaloside IV Inhibits Doxorubicin-Induced Cardiomyocyte Apoptosis Mediated by Mitochondrial Apoptotic Pathway via Activating the PI3K/Akt Pathway. Chem. Pharm. Bull. (Tokyo) 62, 45–53. 10.1248/cpb.c13-00556 24390491

[B56] Jiang Y. Lian Y. J. (2015). Effects of Danhong Injection on Hemodynamics and the Inflammation-Related NF-Κb Signaling Pathway in Patients with Acute Cerebral Infarction. Genet. Mol. Res. 14 (4), 16929–16937. 10.4238/2015 26681040

[B57] Jiang Y. F. Liu Z. Q. Cui W. Zhang W. T. Gong J. P. Wang X. M. (2015). Antioxidant Effect of Salvianolic Acid B on Hippocampal CA1 Neurons in Mice with Cerebral Ischemia and Reperfusion Injury. Chin. J. Integr. Med. 21 (7), 516–522. 10.1007/s11655-014-1791-1 25081897

[B58] Kim J. Y. Kawabori M. Yenari M. A. (2014). Innate Inflammatory Responses in Stroke: Mechanisms and Potential Therapeutic Targets. Curr. Med. Chem. 21, 2076–2097. 10.2174/0929867321666131228205146 24372209 PMC4104826

[B59] Kopáni M. Celec P. Danisovic L. Michalka P. Biró C. (2006). Oxidative Stress and Electron Spin Resonance. Clin. Chim. Acta 364, 61–66. 10.1016/j.cca.2005.05.016 16125687

[B60] Kovacs S. B. Miao E. A. (2017). Gasdermins: Effectors of Pyroptosis. Trends Cel Biol 27, 673–684. 10.1016/j.tcb.2017.05.005 PMC5565696 28619472

[B61] Kyriakis J. M. Avruch J. (2012). Mammalian Mapk Signal Transduction Pathways Activated by Stress and Inflammation: A 10-Year Update. Physiol. Rev. 92, 689–737. 10.1152/physrev.00028.2011 22535895

[B205] Lai X. Cao K. Kong L. Liu Q. Gao Y. (2017). XMAS study investigators. Xingnaojing for Moderate-to-severe Acute ischemic Stroke (XMAS): study protocol for a randomized controlled trial. Trials 18(1), 478–124. 10.1186/s13063-017-2222-y 29037226 PMC5644245

[B62] Lambertsen K. L. Finsen B. Clausen B. H. (2019). Post-stroke Inflammation-Target or Tool for Therapy? Acta Neuropathol. 137, 693–714. 10.1007/s00401-018-1930-z 30483945 PMC6482288

[B63] Lan B. Ge J. W. Cheng S. W. Zheng X. L. Liao J. He C. (2020). Extract of Naotaifang, a Compound Chinese Herbal Medicine, Protects Neuron Ferroptosis Induced by Acute Cerebral Ischemia in Rats. J. Integr. Med. 18, 344–350. 10.1016/j.joim.2020.01.008 32107172

[B64] Lei P. Bai T. Sun Y. (2019). Mechanisms of Ferroptosis and Relations with Regulated Cell Death: A Review. Front. Physiol. 10, 139. 10.3389/fphys.2019.00139 30863316 PMC6399426

[B65] Li F. Li W. Li X. Li F. Zhang L. Wang B. (2016). Geniposide Attenuates Inflammatory Response by Suppressing P2Y14 Receptor and Downstream ERK1/2 Signaling Pathway in Oxygen and Glucose Deprivation-Induced Brain Microvascular Endothelial Cells. J. Ethnopharmacol 185, 77–86. 10.1016/j.jep.2016.03.025 26976766

[B66] Li J. Cao F. Yin H. L. Huang Z. J. Lin Z. T. Mao N. (2020). Ferroptosis: Past, Present and Future. Cell Death Dis 11, 88. 10.1038/s41419-020-2298-2 32015325 PMC6997353

[B67] Li L. Zhang X. Cui L. Wang L. Liu H. Ji H. (2013). Ursolic Acid Promotes the Neuroprotection by Activating Nrf2 Pathway after Cerebral Ischemia in Mice. Brain Res. 1497, 32–39. 10.1016/j.brainres.2012.12.032 23276496

[B68] Li L. M. Zheng B. Zhang R. N. Jin L. S. Zheng C. Y. Wang C. (2015). Chinese Medicine Tongxinluo Increases Tight junction Protein Levels by Inducing KLF5 Expression in Microvascular Endothelial Cells. Cell Biochem Funct 33 (4), 226–234. 10.1002/cbf.3108 25907265

[B69] Li M. H. Ruan L. Y. Chen C. Xing Y. X. Hong W. Du R. H. (2018). Protective Effects of Polygonum Multiflorum on Ischemic Stroke Rat Model Analysed by 1H NMR Metabolic Profiling. J. Pharm. Biomed. Anal. 155, 91–103. 10.1016/j.jpba.2018.03.049 29625260

[B70] Li W. H. Yang Y. L. Cheng X. Liu M. Zhang S. S. Wang Y. H. (2020). Baicalein Attenuates Caspase-independent Cells Death via Inhibiting PARP-1 Activation and AIF Nuclear Translocation in Cerebral Ischemia/reperfusion Rats. Apoptosis 25, 354–369. 10.1007/s10495-020-01600-w 32338336

[B71] Li X. Huang L. Liu G. Fan W. Li B. Liu R. (2020). Ginkgo Diterpene Lactones Inhibit Cerebral Ischemia/reperfusion Induced Inflammatory Response in Astrocytes via TLR4/NF-Κb Pathway in Rats. J. Ethnopharmacol 249, 112365. 10.1016/j.jep.2019.112365 31678414

[B72] Li Y. Ren M. Wang J. Ma R. Chen H. Xie Q. (2021). Progress in Borneol Intervention for Ischemic Stroke: A Systematic Review. Front. Pharmacol. 12, 606682. 10.3389/fphar.2021.606682 34017247 PMC8129537

[B73] Li Y. Yang Y. Zhao Y. Zhang J. Liu B. Jiao S. (2019). Astragaloside IV Reduces Neuronal Apoptosis and Parthanatos in Ischemic Injury by Preserving Mitochondrial Hexokinase-II. Free Radic. Biol. Med. 131, 251–263. 10.1016/j.freeradbiomed.2018.11.033 30502455

[B75] Liao J. Wei B. Chen H. Liu Y. Wang J. (2016). Bioinformatics Investigation of Therapeutic Mechanisms of Xuesaitong Capsule Treating Ischemic Cerebrovascular Rat Model with Comparative Transcriptome Analysis. Am. J. Transl Res. 8, 2438–2449. Available at: https://www.ncbi.nlm.nih.gov/pubmed/27347353 . 27347353 PMC4891458

[B76] Liao J. Xia X. Wang G. Z. Shi Y. M. Ge J. W. (2015). Naotaifang Extract Treatment Results in Increased Ferroportin Expression in the hippocampus of Rats Subjected to Cerebral Ischemia. Mol. Med. Rep. 11, 4047–4052. 10.3892/mmr.2015.3309 25672910 PMC4394947

[B78] Liu B. Li F. Shi J. Yang D. Deng Y. Gong Q. (2016). Gastrodin Ameliorates Subacute Phase Cerebral Ischemia-reperfusion I-njury by I-nhibiting I-nflammation and A-poptosis in R-ats. Mol. Med. Rep. 14 (5), 4144–4152. 10.3892/mmr.2016.5785 27748849 PMC5101922

[B79] Liu D. Dong Z. Xiang F. Liu H. Wang Y. Wang Q. (2020). Dendrobium Alkaloids Promote Neural Function after Cerebral Ischemia-Reperfusion Injury through Inhibiting Pyroptosis Induced Neuronal Death in Both *In Vivo* and *In Vitro* Models. Neurochem. Res. 45, 437–454. 10.1007/s11064-019-02935-w 31865520

[B80] Liu H. Yan Y. Pang P. Mao J. Hu X. Li D. (2019). Angong Niuhuang Pill as Adjuvant Therapy for Treating Acute Cerebral Infarction and Intracerebral Hemorrhage: A Meta-Analysis of Randomized Controlled Trials. J. Ethnopharmacol 237 (237), 307–313. 10.1016/j.jep.2019.03.043 30910581

[B81] Liu R. Yu X. Zhang L. Zhang H. Gong Y. Wu K. (2020). Computed Tomography (CT) Imaging Evaluation of Integrated Traditional Chinese Medicine Cooperative Therapy in Treating Acute Cerebral Infarction: A Randomized Controlled Trial. Medicine (Baltimore) 99, e19998. 10.1097/MD.0000000000019998 32358375 PMC7440100

[B82] Liu S. Y. Cai W. Wang F. Liu Y. Shang Z. P. Zhang X. P. (2017). UHPLC-LTQ-Orbitrap-based Metabolomics Coupled with Metabolomics Pathway Analysis Method for Exploring the protection Mechanism of Kudiezi Injection in a Rat Anti-ischemic Cerebral Reperfusion Damage Model. Chin. J. Nat. Med. 15, 955–960. 10.1016/S1875-5364(18)30013-X 29329654

[B83] Liu X. Chen X. Zhu Y. Wang K. Wang Y. (2017). Effect of Magnolol on Cerebral Injury and Blood Brain Barrier Dysfunction Induced by Ischemia-Reperfusion *In Vivo* and *In Vitro* . Metab. Brain Dis. 32, 1109–1118. 10.1007/s11011-017-0004-6 28378105

[B84] Liu X. (2017). Network Pharmacology-Based Study on Action Mechanism of Ypf Power Treating for Asthma. Chengdu, China: Southwest Jiaotong University.

[B85] Liu Y. Gao J. Peng M. Meng H. Ma H. Cai P. (2018). A Review on Central Nervous System Effects of Gastrodin. Front. Pharmacol. 9, 24. 10.3389/fphar.2018.00024 29456504 PMC5801292

[B86] Liu Y. Tang Q. Shao S. Chen Y. Chen W. Xu X. (2017). Lyophilized Powder of Catalpol and Puerarin Protected Cerebral Vessels from Ischemia by its Anti-apoptosis on Endothelial Cells. Int. J. Biol. Sci. 13, 327–338. 10.7150/ijbs.17751 28367097 PMC5370440

[B87] Liu Z. Gan L. Xu Y. Luo D. Ren Q. Wu S. (2017). Melatonin Alleviates Inflammasome-Induced Pyroptosis through Inhibiting NF-Κb/GSDMD Signal in Mice Adipose Tissue. J. Pineal Res. 63, 63. 10.1111/jpi.12414 28398673

[B88] Long J. Gao M. Kong Y. Shen X. Du X. Son Y. O. (2012). Cardioprotective Effect of Total Paeony Glycosides against Isoprenaline-Induced Myocardial Ischemia in Rats. Phytomedicine 19, 672–676. 10.1016/j.phymed.2012.03.004 22483552

[B91] Ma C. Wang X. Xu T. Yu X. Zhang S. Liu S. (2019). Qingkailing Injection Ameliorates Cerebral Ischemia-Reperfusion Injury and Modulates the AMPK/NLRP3 Inflammasome Signalling Pathway. BMC Complement. Altern. Med. 19, 320. 10.1186/s12906-019-2703-5 31747940 PMC6868863

[B92] Ma C. Wang X. Xu T. Zhang S. Liu S. Zhai C. (2020). An Integrative Pharmacology-Based Analysis of Refined Qingkailing Injection against Cerebral Ischemic Stroke: A Novel Combination of Baicalin, Geniposide, Cholic Acid, and Hyodeoxycholic Acid. Front. Pharmacol. 11 (11), 519. 10.3389/fphar.2020.00519 32457601 PMC7227481

[B93] Ma Y. Wang W. Yang J. Zhang S. Li Z. Li F. (2020). A Network Pharmacology Technique to Investigate the Synergistic Mechanisms of Salvia Miltiorrhiza and Radix Puerariae in Treatment of Cardio-Cerebral Vascular Diseases. Evid. Based Complement. Alternat Med. 2020, 6937186. 10.1155/2020/6937186 33082828 PMC7566220

[B94] Manwani B. McCullough L. D. (2013). Function of the Master Energy Regulator Adenosine Monophosphate-Activated Protein Kinase in Stroke. J. Neurosci. Res. 91, 1018–1029. 10.1002/jnr.23207 23463465 PMC4266469

[B95] Maugeri R. Schiera G. Di Liegro C. M. Fricano A. Iacopino D. G. Di Liegro I. (2016). Aquaporins and Brain Tumors. Int. J. Mol. Sci. 17, 17. 10.3390/ijms17071029 PMC4964405 27367682

[B96] Mei Z. G. Tan L. J. Wang J. F. Li X. L. Huang W. F. Zhou H. J. (2017). Fermented Chinese Formula Shuan-Tong-Ling Attenuates Ischemic Stroke by Inhibiting Inflammation and Apoptosis. Neural Regen. Res. 12, 425–432. 10.4103/1673-5374.202946 28469657 PMC5399720

[B97] Meng H. Jin W. Yu L. Xu S. Wan H. He Y. (2021). Protective Effects of Polysaccharides on Cerebral Ischemia: A Mini-Review of the Mechanisms. Int. J. Biol. Macromol 169, 463–472. 10.1016/j.ijbiomac10.1016/j.ijbiomac.2020.12.124 33347928

[B98] Miao M. S. Guo L. Li R. Q. Zhang X. L. (2016). Radix Ilicis Pubescentis Total Flavonoids Ameliorates Neuronal Damage and Reduces Lesion Extent in a Mouse Model of Transient Ischemic Attack. Neural Regen. Res. 11, 441–446. 10.4103/1673-5374.179056 27127483 PMC4829009

[B99] Mizushima N. Komatsu M. (2011). Autophagy: Renovation of Cells and Tissues. Cell 147, 728–741. 10.1016/j.cell.2011.10.026 22078875

[B100] Mizushima N. (2010). The Role of the Atg1/ULK1 Complex in Autophagy Regulation. Curr. Opin. Cel Biol 22, 132–139. 10.1016/j.ceb.2009.12.004 20056399

[B101] Moritz M. Pfeifer S. Balmayor E. R. Mittermayr R. Wolbank S. Redl H. (2017). VEGF Released from a Fibrin Biomatrix Increases VEGFR-2 Expression and Improves Early Outcome after Ischaemia-Reperfusion Injury. J. Tissue Eng. Regen. Med. 11, 2153–2163. 10.1002/term.2114 26777435

[B102] Nan L. Xie Q. Chen Z. Zhang Y. Chen Y. Li H. (2020). Involvement of PARP-1/AIF Signaling Pathway in Protective Effects of Gualou Guizhi Decoction against Ischemia-Reperfusion Injury-Induced Apoptosis. Neurochem. Res. 45 (2), 278–294. 10.1007/s11064-019-02912-3 31792665

[B103] Navarro-Yepes J. Zavala-Flores L. Anandhan A. Wang F. Skotak M. Chandra N. (2014). Antioxidant Gene Therapy against Neuronal Cell Death. Pharmacol. Ther. 142, 206–230. 10.1016/j.pharmthera.2013.12.007 24333264 PMC3959583

[B104] Nederlof R. Eerbeek O. Hollmann M. W. Southworth R. Zuurbier C. J. (2014). Targeting Hexokinase II to Mitochondria to Modulate Energy Metabolism and Reduce Ischaemia-Reperfusion Injury in Heart. Br. J. Pharmacol. 171, 2067–2079. 10.1111/bph.12363 24032601 PMC3976622

[B105] O'Connell G. C. Chantler P. D. Barr T. L. (2017). Stroke-associated Pattern of Gene Expression Previously Identified by Machine-Learning Is Diagnostically Robust in an Independent Patient Population. Genom Data 14, 47–52. 10.1016/j.gdata.2017.08.006 28932682 PMC5596252

[B106] Olivier M. Asmis R. Hawkins G. A. Howard T. D. Cox L. A. (2019). The Need for Multi-Omics Biomarker Signatures in Precision Medicine. Int. J. Mol. Sci. 20 (19), 4781. 10.3390/ijms20194781 PMC6801754 31561483

[B107] Olmez I. Ozyurt H. (2012). Reactive Oxygen Species and Ischemic Cerebrovascular Disease. Neurochem. Int. 60, 208–212. 10.1016/j.neuint.2011.11.009 22122807

[B108] Ong W. Y. Farooqui T. Koh H. L. Farooqui A. A. Ling E. A. (2015). Protective Effects of Ginseng on Neurological Disorders. Front. Aging Neurosci. 7, 129–142. 10.3389/fnagi.2015.00129 26236231 PMC4503934

[B109] Orgah J. O. Ren J. Liu X. Orgah E. A. Gao X. M. Zhu Y. (2019). Danhong Injection Facilitates Recovery of post-stroke Motion Deficit via Parkin-Enhanced Mitochondrial Function. Restor Neurol. Neurosci. 37 (4), 375–395. 10.3233/RNN-180828 31282440

[B110] Orrenius S. Zhivotovsky B. Nicotera P. (2003). Regulation of Cell Death: the Calcium-Apoptosis Link. Nat. Rev. Mol. Cel Biol 4, 552–565. 10.1038/nrm1150 12838338

[B111] Pan R. Cai J. Zhan L. Guo Y. Huang R. Y. Li X. (2017). Buyang Huanwu Decoction Facilitates Neurorehabilitation through an Improvement of Synaptic Plasticity in Cerebral Ischemic Rats. BMC Complement. Altern. Med. 17 (1), 173. 10.1186/s12906-017-1680-9 28351388 PMC5371213

[B112] Pan X. W. Wang M. J. Gong S. S. Sun M. H. Wang Y. Zhang Y. Y. (2020). YiQiFuMai Lyophilized Injection Ameliorates tPA-Induced Hemorrhagic Transformation by Inhibiting Cytoskeletal Rearrangement Associated with ROCK1 and NF-kappaB Signaling Pathways. J. Ethnopharmacol 262, 113161. 10.1016/j.jep.2020.113161 32730882

[B113] Peng Z. Wang S. Chen G. Cai M. Liu R. Deng J. (2015). Gastrodin Alleviates Cerebral Ischemic Damage in Mice by Improving Anti-oxidant and Anti-inflammation Activities and Inhibiting Apoptosis Pathway. Neurochem. Res. 40, 661–673. 10.1007/s11064-015-1513-5 25582916

[B114] Pereira E. R. Frudd K. Awad W. Hendershot L. M. (2014). Endoplasmic Reticulum (ER) Stress and Hypoxia Response Pathways Interact to Potentiate Hypoxia-Inducible Factor 1 (HIF-1) Transcriptional Activity on Targets like Vascular Endothelial Growth Factor (VEGF). J. Biol. Chem. 289, 3352–3364. 10.1074/jbc.M113.507194 24347168 PMC3916539

[B115] Qiu Y. Cao Y. Cao W. Jia Y. Lu N. (2020). The Application of Ferroptosis in Diseases. Pharmacol. Res. 159, 104919. 10.1016/j.phrs.2020.104919 32464324

[B116] Rai A. T. Seldon A. E. Boo S. Link P. S. Domico J. R. Tarabishy A. R. (2016). A Population-Based Incidence of Acute Large Vessel Occlusions and Thrombectomy Eligible Patients Indicates Significant Potential for Growth of Endovascular Stroke Therapy in the USA. J. Neurointerv Surg. 9 (8), 722–726. 10.1136/neurintsurg-2016-012515 27422968 PMC5583675

[B117] Robinson N. Ganesan R. Hegedus C. Kovacs K. Kufer T. A. Virag L. (2019). Programmed Necrotic Cell Death of Macrophages: Focus on Pyroptosis, Necroptosis, and Parthanatos. Redox Biol. 26, 101239. 10.1016/j.redox.2019.101239 31212216 PMC6582207

[B118] Santa-Cecilia F. V. Socias B. Ouidja M. O. Sepulveda-Diaz J. E. Acuna L. Silva R. L. (2016). Doxycycline Suppresses Microglial Activation by Inhibiting the P38 MAPK and NF-kB Signaling Pathways. Neurotox Res. 29, 447–459. 10.1007/s12640-015-9592-2 26745968

[B119] Shen J. Huang K. Zhu Y. Xu K. Zhan R. Pan J. (2020). Buyang Huanwu Decoction Promotes Angiogenesis after Cerebral Ischemia by Inhibiting the Nox4/ROS Pathway. Evid. Based Complement. Alternat Med. 2020, 5264205. 10.1155/2020/5264205 32802129 PMC7415092

[B120] Shi A. Xiang J. He F. Zhu Y. Zhu G. Lin Y. (2018). The Phenolic Components of Gastrodia Elata Improve Prognosis in Rats after Cerebral Ischemia/Reperfusion by Enhancing the Endogenous Antioxidant Mechanisms. Oxid Med. Cel Longev 2018, 7642158. 10.1155/2018/7642158 PMC5885496 29765502

[B121] Shi J. Zhao Y. Wang K. Shi X. Wang Y. Huang H. (2015). Cleavage of GSDMD by Inflammatory Caspases Determines Pyroptotic Cell Death. Nature 526, 660–665. 10.1038/nature15514 26375003

[B122] Son T. G. Camandola S. Arumugam T. V. Cutler R. G. Telljohann R. S. Mughal M. R. (2010). Plumbagin, a Novel Nrf2/ARE Activator, Protects against Cerebral Ischemia. J. Neurochem. 112, 1316–1326. 10.1111/j.1471-4159.2009.06552.x 20028456 PMC2819586

[B123] Sonkusare S. K. Bonev A. D. Ledoux J. Liedtke W. Kotlikoff M. I. Heppner T. J. (2012). Elementary Ca2+ Signals through Endothelial TRPV4 Channels Regulate Vascular Function. Science 336, 597–601. 10.1126/science.1216283 22556255 PMC3715993

[B124] Subedi L. Gaire B. P. (2021). Phytochemicals as Regulators of Microglia/macrophages Activation in Cerebral Ischemia. Pharmacol. Res. 165, 105419. 10.1016/j.phrs.2021.105419 33450385

[B125] Sun C. Lai X. Huang X. Zeng Y. (2014). Protective Effects of Ginsenoside Rg1 on Astrocytes and Cerebral Ischemic-Reperfusion Mice. Biol. Pharm. Bull. 37 (12), 1891–1898. 10.1248/bpb.b14-00394 25451838

[B126] Sun Y. J. Zhang N. N. Zhang M. (2019). Effect of Resveratrol on Cellular Pyrolysisof Rat Brain during Ischemia Reperfusion and on NLRP3 Inflammatory Bodies, Caspase-1 and ZO-1 in Microglia. J. Hainan Med. Univ. 25 (17), 1291–1294. 10.13210/j.cnki.jhmu.20190716.002

[B127] Szarka N. Pabbidi M. R. Amrein K. Czeiter E. Berta G. Pohoczky K. (2018). Traumatic Brain Injury Impairs Myogenic Constriction of Cerebral Arteries: Role of Mitochondria-Derived H2O2 and TRPV4-dependent Activation of BKca Channels. J. Neurotrauma 35, 930–939. 10.1089/neu.2017.5056 29179622 PMC5865628

[B128] Tan L. Wang Y. Jiang Y. Wang R. Zu J. Tan R. (2020). Hydroxysafflor Yellow A Together with Blood–Brain Barrier Regulator Lexiscan for Cerebral Ischemia Reperfusion Injury Treatment. ACS Omega 5, 19151–19164. 10.1021/acsomega.0c02502 32775917 PMC7408215

[B129] Tsoi B. Chen X. Gao C. Wang S. Yuen S. C. Yang D. (2019a). Neuroprotective Effects and Hepatorenal Toxicity of Angong Niuhuang Wan against Ischemia-Reperfusion Brain Injury in Rats. Front. Pharmacol. 10, 593. 10.3389/fphar.2019.00593 31191319 PMC6548905

[B130] Tsoi B. Wang S. Gao C. Luo Y. Li W. Yang D. (2019b). Realgar and Cinnabar Are Essential Components Contributing to Neuroprotection of Angong Niuhuang Wan with No Hepatorenal Toxicity in Transient Ischemic Brain Injury. Toxicol. Appl. Pharmacol. 377, 114613. 10.1016/j.taap.2019.114613 31207256

[B131] Vela L. Gonzalo O. Naval J. Marzo I. (2013). Direct Interaction of Bax and Bak Proteins with Bcl-2 Homology Domain 3 (BH3)-Only Proteins in Living Cells Revealed by Fluorescence Complementation. J. Biol. Chem. 288, 4935–4946. 10.1074/jbc.M112.422204 23283967 PMC3576097

[B133] Wang G. J. Zhang L. C. Chen B. Zhang Y. Y. Kong L. Han J. (2019). Protective Effects of Salvianolic Acid B on Cerebral Ischemic Reperfusion Injury in Rats. Chin. Arch. Trad Med. 37 (07), 1566–1568. 10.13193/j.issn.1673-7717.2019.07.006

[B134] Wang L. Yuan Q. Marshall G. Cui X. Cheng L. Li Y. (2010). Adverse drug reactions and adverse events of 33 varieties of traditional Chinese medicine injections on National Essential medicines List (2004 edition) of China: an overview on published literatures. J Evid Based Med. 32(2), 95–104. 10.1111/j.1756-5391.2010.01073.x 21349051

[B135] Wang M. Wang J. Liu Z. (2018). Efects of Intermedin on Autophagy in Cerebral Ischemia/reperfusion Injury. Neuropeptides 68, 15–21. 10.1016/j.npep.2017.10.004 29128104

[B136] Wang M. Yao M. Liu J. Takagi N. Yang B. Zhang M. (2020a). Ligusticum Chuanxiong Exerts Neuroprotection by Promoting Adult Neurogenesis and Inhibiting Inflammation in the hippocampus of ME Cerebral Ischemia Rats. J. Ethnopharmacol 249, 112385. 10.1016/j.jep.2019.112385 31730888

[B137] Wang P. Shao B. Z. Deng Z. Chen S. Yue Z. Miao C. Y. (2018). Autophagy in Ischemic Stroke. Prog. Neurobiol. 163, 98–117. 10.1016/j.pneurobio.2018.01.001 29331396

[B138] Wang P. R. Wang J. S. Zhang C. Song X. F. Tian N. Kong L. Y. (2013). Decotion Induced Protective Autophagy against the Injury of Cerebral Ischemia/reperfusion via MAPK-mTOR Signaling Pathway. J. Ethnopharmacol 149, 270–280. 10.1016/j.jep.2013.06.035 23811213

[B139] Wang R. Shi L. Liu S. Liu Z. Song F. Sun Z. (2019). Mass Spectrometry-Based Urinary Metabolomics for the Investigation on the Mechanism of Action of Eleutherococcus Senticosus (Rupr. & Maxim.) Maxim. Leaves against Ischemic Stroke in Rats. J. Ethnopharmacol 241, 111969. 10.1016/j.jep.2019.111969 31125596

[B140] Wang S. Fu J. L. Hao H. F. Jiao Y. N. Li P. P. Han S. Y. (2021). Metabolic Reprogramming by Traditional Chinese Medicine and its Role in Effective Cancer Therapy. Pharmacol. Res. 170, 105728. 10.1016/j.phrs.2021.105728 34119622

[B141] Wang S. B. Pang X. B. Zhao Y. Wang Y. H. Zhang L. Yang X. Y. (2012). Protection of Salvianolic Acid A on Rat Brain from Ischemic Damage via Soluble Epoxide Hydrolase Inhibition. J. Asian Nat. Prod. Res. 14 (11), 1084–1092. 10.1080/10286020.2012.723200 23106500

[B142] Wang S. F. (2017). The Therapeutic Effect and Mechanism of Mu-Xiang-You-Formulation on Cerebral Ischemia-Reperfusion Injury in Rats. Ningxia, China: Master’s Degree of Ningxia Medical University.

[B143] Wang Y. An R. Umanah G. K. Park H. Nambiar K. Eacker S. M. (2016). A Nuclease that Mediates Cell Death Induced by DNA Damage and poly(ADP-Ribose) Polymerase-1. Science 354, 6308. 10.1126/science.aad6872 PMC5134926 27846469

[B144] Wang Y. Kim N. S. Haince J. F. (2011). Poly(ADP-ribose) (PAR) Binding to Apoptosis-Inducing Factor Is Critical for PAR Polymerase-1-dependent Cell Death (Parthanatos). Sci. signaling 4 (167), ra20. 10.1126/scisignal.2000902 PMC3086524 21467298

[B145] Wang Y. Shi Y. Zou J. Zhang X. Wang M. Guo D. (2020b). The Intranasal Administration of Carthamus tinctorius L. Extract/phospholipid Complex in the Treatment of Cerebral Infarction via the TNF-Kβ/MAPK Pathway. Biomed. Pharmacother. 130, 110563. 10.1016/j.biopha.2020.110563 32745908

[B146] Wang Y. Xiao G. He S. Liu X. Zhu L. Yang X. (2020c). Protection against Acute Cerebral Ischemia/reperfusion Injury by QiShenYiQi via Neuroinflammatory Network Mobilization. Biomed. Pharmacother. 125, 109945. 10.1016/j.biopha.2020.109945 32028240

[B147] Wang Y. Y. (2016). Internal Medicine of Traditional Chinese Medicine [M]. Shanghai: Shanghai Science and Technology Press, 124–132.

[B148] Wang Z. Yuan Y. Zhang Z. Ding K. (2019). Inhibition of miRNA-27b Enhances Neurogenesis via AMPK Activation in a Mouse Ischemic Stroke Model. FEBS Open Bio 9, 859–869. 10.1002/2211-5463.12614 PMC6487723 30974042

[B149] Wu C. J C. J. Chen J. T. Yen T. L. Jayakumar T. Chou D. S. Hsiao G. (2011). Neuroprotection by the Traditional Chinese Medicine, Tao-Hong-Si-Wu-Tang, against Middle Cerebral Artery Occlusion-Induced Cerebral Ischemia in Rats. Evid. Based Complement. Alternat Med. 2011, 803015. 10.1155/2011/803015 21076527 PMC2975073

[B150] Wu H. R. (2018). Study on the Regulating Effect of Taohong Siwu Decoction on Brain-Derived Neurotrophic Factor in Rats with Cerebral Ischemia Reperfusion. Hefei, China: Degree. Anhui University of Chinese Medicine.

[B151] Wu J. Zhang X. Zhang B. (2014). Qingkailing Injection for the Treatment of Acute Stroke: a Systematic Review and Meta-Analysis. J. Tradit Chin. Med. 34 (2), 131–139. 10.1016/s0254-6272(14)60066-2 24783921

[B152] Wu L. Li F. Zhao C. Ming Y. Zheng C. Li Y. (2020). Effects and Mechanisms of Traditional Chinese Herbal Medicine in the Treatment of Ischemic Cardiomyopathy. Pharmacol. Res. 151, 104488. 10.1016/j.phrs.2019.104488 31734344

[B153] Wu Q. Q. Wang Y. Senitko M. Meyer C. Wigley W. C. Ferguson D. A. (2011). Bardoxolone Methyl (BARD) Ameliorates Ischemic AKI and Increases Expression of Protective Genes Nrf2, PPARgamma, and HO-1. Am. J. Physiol. Ren. Physiol 300, F1180–F1192. 10.1152/ajprenal.00353.2010 PMC3094059 21289052

[B155] Xiang Y. Yao X. Wang X. Zhao H. Zou H. Wang L. (2019). Houshiheisan Promotes Angiogenesis via HIF-1alpha/VEGF and SDF-1/CXCR4 Pathways: *In Vivo* and *In Vitro* . Biosci. Rep. 39. 10.1042/BSR20191006 PMC6822506 31652450

[B156] Xie W. Wang X. Xiao T. Cao Y. Wu Y. Yang D. (2021). Protective Effects and Network Analysis of Ginsenoside Rb1 against Cerebral Ischemia Injury: A Pharmacological Review. Front. Pharmacol. 2 (12), 604811. 10.3389/fphar.2021.604811 PMC8283782 34276353

[B157] Xie Y. Hou W. Song X. Yu Y. Huang J. Sun X. (2016). Ferroptosis: Process and Function. Cell Death Differ 23, 369–379. 10.1038/cdd.2015.158 26794443 PMC5072448

[B158] Xu A. L. Zheng G. Y. Wang Z. J. Chen X. D. Jiang Q. (2016). Neuroprotective Effects of Ilexonin A Following Transient Focal Cerebral Ischemia in Rats. Mol. Med. Rep. 13 (4), 2957–2966. 10.3892/mmr.2016.4921 26936330 PMC4805093

[B159] Xu L. (2019). Research on the Mechanism of Tanshinone IIA Inhibiting Ferroptosis by Regulating Iron Homeostasis in the Model of Cerebral Ischemia. Master Dissertation of Anhui medical university. Anhui Medical University, Hefei. Available at: https://kns.cnki.net/kns8/defaultresult/index .

[B160] Xu Z. Liu W. Huang H. (2020). Astragaloside IV Alleviates Cerebral Ischemia-Reperfusion Injury by Activating the Janus Kinase 2 and Signal Transducer and Activator of Transcription 3 Signaling Pathway. Pharmacology 105 (3-4), 181–189. 10.1159/000503361 31825924

[B161] Yang B. Sun Y. Lv C. Zhang W. Chen Y. (2020). Procyanidins Exhibits Neuroprotective Activities against Cerebral Ischemia Reperfusion Injury by Inhibiting TLR4-NLRP3 Inflammasome Signal Pathway. Psychopharmacology (Berl) 237, 3283–3293. 10.1007/s00213-020-05610-z 32729095

[B162] Yang C. M. Chen L. D. Tao J. (2012). The Ancient Hinduists in Gualou Guizhi Decoction. Liaoning Zhongyi Zazhi 39 (08), 166–167.

[B163] Yang H. Li L. Zhou K. Wang Y. Guan T. Chai C. (2016). Shengmai Injection Attenuates the Cerebral Ischemia/reperfusion Induced Autophagy via Modulation of the AMPK, mTOR and JNK Pathways. Pharm. Biol. 54, 2288–2297. 10.3109/13880209.2016.1155625 26983890

[B165] Yang Y. Rosenberg G. A. (2011). Blood-brain Barrier Breakdown in Acute and Chronic Cerebrovascular Disease. Stroke 42, 3323–3328. 10.1161/STROKEAHA.110.608257 21940972 PMC3584169

[B166] Yao X. Uchida K. Papadopoulos M. C. Zador Z. Manley G. T. Verkman A. S. (2015). Mildly Reduced Brain Swelling and Improved Neurological Outcome in Aquaporin-4 Knockout Mice Following Controlled Cortical Impact Brain Injury. J. Neurotrauma 32, 1458–1464. 10.1089/neu.2014.3675 25790314 PMC4589265

[B167] Ye Y. Li J. Cao X. Chen Y. Ye C. Chen K. (2016). Protective Effect of N-Butyl Alcohol Extracts from Rhizoma Pinelliae Pedatisectae against Cerebral Ischemia-Reperfusion Injury in Rats. J. Ethnopharmacol 188, 259–265. 10.1016/j.jep.2016.04.046 27132713

[B168] Yu F. Xue W. Dong L. Hu X. Huang D. Wang K. (2019). Tetrahydroxystilbene Glucoside Suppresses NAPDH Oxidative Stress to Mitigate Apoptosis and Autophagy Induced by Cerebral Ischemia/Reperfusion Injury in Mice. Evid. Based Complement. Alternat Med. 2019, 3913981. 10.1155/2019/3913981 31379960 PMC6662418

[B169] Zeng C. Wang R. Tan H. (2019). Role of Pyroptosis in Cardiovascular Diseases and its Therapeutic Implications. Int. J. Biol. Sci. 15, 1345–1357. 10.7150/ijbs.33568 31337966 PMC6643148

[B172] Zhang D. S. Fu M. D. Song C. L. Wang C. Y. Lin X. C. Liu Y. L. (2012). Expressions of Apoptosis-Related Proteins in Rats with Focal Cerebral Ischemia after Angong Niuhuang Sticker point Application. Neural Regen. Res. 7 (30), 2347–2353. 10.3969/j.issn.1673-5374.2012.30.004 25538759 PMC4268739

[B173] Zhang D. S. Liu Y. L. Zhu D. Q. Huang X. J. Luo C. H. (2015). Point Application with Angong Niuhuang Sticker Protects Hippocampal and Cortical Neurons in Rats with Cerebral Ischemia. Neural Regen. Res. 10 (2), 286–291. 10.4103/1673-5374.152384 25883629 PMC4392678

[B174] Zhang H. Song Y. Feng C. (2020). Improvement of Cerebral Ischemia/reperfusion Injury by Daucosterol Palmitate-Induced Neuronal Apoptosis Inhibition via PI3K/Akt/mTOR Signaling Pathway. Metab. Brain Dis. 35, 1035–1044. 10.1007/s11011-020-00575-6 32363473

[B175] Zhang H. Zhai L. Wang T. Li S. Guo Y. (2017). Picroside II Exerts a Neuroprotective Effect by Inhibiting the Mitochondria Cytochrome C Signal Pathway Following Ischemia Reperfusion Injury in Rats. J. Mol. Neurosci. 61, 267–278. 10.1007/s12031-016-0870-0 28054226

[B176] Zhang J. Jiang Y. Liu N. Shen T. Jung H. W. Liu J. (2019a). A Network-Based Method for Mechanistic Investigation and Neuroprotective Effect on Post-treatment of Senkyunolid-H against Cerebral Ischemic Stroke in Mouse. Front. Neurol. 10, 1299. 10.3389/fneur.2019.01299 31920923 PMC6930873

[B177] Zhang J. Liu M. Huang M. Chen M. Zhang D. Luo L. (2019b). Ginsenoside F1 Promotes Angiogenesis by Activating the IGF-1/IGF1R Pathway. Pharmacol. Res. 144, 292–305. 10.1016/j.phrs.2019.04.021 31048033

[B178] Zhang J. K J. K. Yang L. Meng G. L. Fan J. Chen J. Z. He Q. Z. (2012). Protective Effect of Tetrahydroxystilbene Glucoside against Hydrogen Peroxide-Induced Dysfunction and Oxidative Stress in Osteoblastic MC3T3-E1 Cells. Eur. J. Pharmacol. 689, 31–37. 10.1016/j.ejphar.2012.05.045 22683865

[B179] Zhang J. Y. (2019). Study on the Mechanism of Resveratrol in Promoting Peripheral Nerve Repair. Doctoral Dissertation of Jilin university. Changchun, China: Jilin university. Available at: https://kns.cnki.net/kns8/defaultresult/index .

[B180] Zhang K. Li Y. J. Yang Q. Gerile O. Yang L. Li X. B. (2013). Neuroprotective Effects of Oxymatrine against Excitotoxicity Partially through Down-Regulation of NR2B-Containing NMDA Receptors. Phytomedicine 20, 343–350. 10.1016/j.phymed.2012.10.018 23219339

[B182] Zhang Q. Bian H. Li Y. Guo L. Tang Y. Zhu H. (2014). Preconditioning with the Traditional Chinese Medicine Huang-Lian-Jie-Du-Tang Initiates HIF-1alpha-dependent Neuroprotection against Cerebral Ischemia in Rats. J. Ethnopharmacol 154, 443–452. 10.1016/j.jep.2014.04.022 24751364

[B183] Zhang Q Q. Zhao H. Wang L. Zhang Q. Wang H. (2012). Effects of Wind-Dispelling Drugs and Deficiency-Nourishing Drugs of Houshiheisan Compound Prescription on Astrocyte Activation and Inflammatory Factor Expression in the Corpus Striatum of Cerebral Ischemia Rats. Neural Regen. Res. 7 (24), 1851–1857. 10.3969/j.issn.1673-5374.2012.24.002 25624810 PMC4298897

[B184] Zhang R. Tang S. Huang W. Liu X. Li G. Chi H. (2015). Protection of the Brain Following Cerebral Ischemia through the Attenuation of PARP-1-Induced Neurovascular Unit Damage in Rats. Brain Res. 1624, 9–18. 10.1016/j.brainres.2015.07.023 26220474

[B185] Zhang S. Wang X. Cheng F. Ma C. Fan S. Xu W. (2020). Network Pharmacology-Based Approach to Revealing Biological Mechanisms of Qingkailing Injection against Ischemic Stroke: Focusing on Blood-Brain Barrier. Evid. Based Complement. Alternat Med. 2020, 2914579. 10.1155/2020/2914579 32908557 PMC7474352

[B186] Zhang S. Zhang Y. Li H. Xu W. Chu K. Chen L. (2015). Antioxidant and Anti-excitotoxicity Effect of Gualou Guizhi Decoction on Cerebral Ischemia/reperfusion Injury in Rats. Exp. Ther. Med. 9 (6), 2121–2126. 10.3892/etm.2015.2386 26136945 PMC4473344

[B187] Zhang W. Zhang Q. Deng W. Li Y. Xing G. Shi X. (2014). Neuroprotective Effect of Pretreatment with Ganoderma Lucidum in Cerebral Ischemia/reperfusion Injury in Rat hippocampus. Neural Regen. Res. 9, 1446–1452. 10.4103/1673-5374.139461 25317156 PMC4192946

[B188] Zhang X. Zhang Y. Tang S. Yu L. Zhao Y. Ren Q. (2018). Pien-Tze-Huang Protects Cerebral Ischemic Injury by Inhibiting Neuronal Apoptosis in Acute Ischemic Stroke Rats. J. Ethnopharmacol 219, 117–125. 10.1016/j.jep.2018.03.018 29550579

[B189] Zhang Y. Fan L. Li H. Wang X. Xu W. Chu K. (2018). Gualou Guizhi Granule Protects against Oxidative Injury by Activating Nrf2/ARE Pathway in Rats and PC12 Cells. Neurochem. Res. 43 (5), 1003–1009. 10.1007/s11064-018-2507-x 29564698

[B190] Zhang Y. Zhang S. Li H. Huang M. Xu W. Chu K. (2015). Ameliorative Effects of Gualou Guizhi Decoction on Inflammation in Focal Cerebral Ischemic-Reperfusion Injury. Mol. Med. Rep. 12 (1), 988–994. 10.3892/mmr.2015.3515 25815894 PMC4438940

[B191] Zhao Q. Cheng X. Wang X. Wang J. Zhu Y. Ma X. (2016). Neuroprotective Effect and Mechanism of Mu-Xiang-You-Fang on Cerebral Ischemia-Reperfusion Injury in Rats. J. Ethnopharmacol 192, 140–147. 10.1016/j.jep.2016.07.016 27396346

[B192] Zhao T. Tang H. Xie L. Zheng Y. Ma Z. Sun Q. (2019). Scutellaria Baicalensis Georgi. (Lamiaceae): a Review of its Traditional Uses, Botany, Phytochemistry, Pharmacology and Toxicology. J. Pharm. Pharmacol. 71 (9), 1353–1369. 10.1111/jphp.13129 31236960

[B193] Zheng X. W. Shan C. S. Xu Q. Q. Wang Y. Shi Y. H. Wang Y. (2018). Buyang Huanwu Decoction Targets SIRT1/VEGF Pathway to Promote Angiogenesis after Cerebral Ischemia/Reperfusion Injury. Front. Neurosci. 12, 911. 10.3389/fnins.2018.00911 30564092 PMC6288378

[B194] Zheng Y. Y. Dong Z. Lu X. Q. Xia Y. P. Zhu S. B. (2015). Analysis on 315 Cases of Clinical Adverse Drug Reaction/event Induced by Gastrodin. Zhongguo Zhong Yao Za Zhi 40 (10), 2037–2041. 26390669

[B195] Zhou D. Cen K. Liu W. Liu F. Liu R. Sun Y. (2021). Xuesaitong Exerts Long-Term Neuroprotection for Stroke Recovery by Inhibiting the ROCKII Pathway, *In Vitro* and *In Vivo* . J. Ethnopharmacol 272, 113943. 10.1016/j.jep.2021.113943 33617967

[B196] Zhou F. Wang L. Liu P. Hu W. Zhu X. Shen H. (2014). Puerarin Protects Brain Tissue against Cerebral Ischemia/reperfusion Injury by Inhibiting the Inflammatory Response. Neural Regen. Res. 9 (23), 2074–2080. 10.4103/1673-5374.147934 25657724 PMC4316472

[B197] Zhou Q. B. Ju X. N. Wang X. Y. Wang M. H. Kong F. Sun C. (2016). Pretreatment with Baicalin Attenuates Hypoxia and Glucose Deprivation-Induced Injury in SH-Sy5y Cells. Chin. J. Integr. Med. 22 (3), 201–206. 10.1007/s11655-015-2326-8 26688183

[B198] Zhou X. Y. Luo Y. Zhu Y. M. Liu Z. H. Kent T. A. Rong J. G. (2017). Inhibition of Autophagy Blocks Cathepsins-tBid-Mitochondrial Apoptotic Signaling Pathway via Stabilization of Lysosomal Membrane in Ischemic Astrocytes. Cel Death Dis 8, e2618. 10.1038/cddis.2017.34 PMC5386481 28206988

[B199] Zhou Y. Li H. Q. Lu L. Fu D. L. Liu A. J. Li J. H. (2014). Ginsenoside Rg1 Provides Neuroprotection against Blood Brain Barrier Disruption and Neurological Injury in a Rat Model of Cerebral Ischemia/reperfusion through Downregulation of Aquaporin 4 Expression. Phytomedicine 21 (7), 998–1003. 10.1016/j.phymed.2013.12.005 24462216

[B200] Zhou Z. Dun L. Wei B. Gan Y. Liao Z. Lin X. (2020). An H, Musk Ketone Induces Neural Stem Cell Proliferation and Differentiation in Cerebral Ischemia via Activation of the PI3K/Akt Signaling Pathway. Neuroscience 435, 1–9. 10.1016/j.neuroscience.2020.02.031 32112919

[B201] Zhou Z. Q. Li Y. L. Ao Z. B. Wen Z. L. Chen Q. W. Huang Z. G. (2017). Baicalin Protects Neonatal Rat Brains against Hypoxic-Ischemic Injury by Upregulating Glutamate Transporter 1 via the Phosphoinositide 3-kinase/protein Kinase B Signaling Pathway. Neural Regen. Res. 12 (10), 1625–1631. 10.4103/1673-5374.217335 29171427 PMC5696843

[B202] Zhu B. Cao H. Sun L. Li B. Guo L. Duan J. (2018). Metabolomics-based Mechanisms Exploration of Huang-Lian Jie-Du Decoction on Cerebral Ischemia via UPLC-Q-TOF/MS Analysis on Rat Serum. J. Ethnopharmacol 216, 147–156. 10.1016/j.jep.2018.01.015 29360497

[B203] Zou H. Long J. Zhang Q. Zhao H. Bian B. Wang Y. (2016). Induced Cortical Neurogenesis after Focal Cerebral Ischemia--Three Active Components from Huang-Lian-Jie-Du Decoction. J. Ethnopharmacol 178, 115–124. 10.1016/j.jep.2015.12.001 26657578

